# Device-measured vigorous intermittent lifestyle physical activity (VILPA) and major adverse cardiovascular events: evidence of sex differences

**DOI:** 10.1136/bjsports-2024-108484

**Published:** 2024-10-28

**Authors:** Emmanuel Stamatakis, Matthew Ahmadi, Raaj Kishore Biswas, Borja del Pozo Cruz, Cecilie Thøgersen-Ntoumani, Marie H Murphy, Angelo Sabag, Scott Lear, Clara Chow, Jason M R Gill, Mark Hamer

**Affiliations:** 1 Mackenzie Wearables Research Hub, Charles Perkins Centre, University of Sydney, Sydney, New South Wales, Australia; 2 School of Health Sciences, Faculty of Medicine and Health, University of Sydney, Sydney, New South Wales, Australia; 3 Faculty of Sports Sciences, Universidad Europea de Madrid, Madrid, Spain; 4 Faculty of Biomedical and Health Sciences, Universidad Europea de Madrid, Madrid, Spain; 5 Department of Sports Science and Clinical Biomechanics, University of Southern Denmark, Odense, Denmark; 6 Danish Centre for Motivation and Behaviour Science, Department of Sports Science and Clinical Biomechanics, University of Southern Denmark, Odense, Syddanmark, Denmark; 7 Physical Activity for Health Research Centre, Moray House School of Education and Sport, University of Edinburgh, Edinburgh, UK; 8 Faculty of Health Sciences, Simon Fraser University, Vancouver, British Columbia, Canada; 9 Westmead Applied Research Centre, University of Sydney and Department of Cardiology, Westmead Hospital, Sydney, New South Wales, Australia; 10 School of Cardiovascular and Metabolic Health, University of Glasgow, Glasgow, UK; 11 Institute of Sport Exercise and Health, Division Surgery Interventional Science, University College London, London, London, UK

**Keywords:** Cardiovascular Diseases, Physical activity, Exercise, Heart disease, Cohort Studies

## Abstract

**ABSTRACT:**

**Background:**

Vigorous intermittent lifestyle physical activity (VILPA) refers to brief bouts of intense physical activity embedded into daily life.

**Objective:**

To examine sex differences in the dose–response association of VILPA with major adverse cardiovascular events (MACE) and its subtypes.

**Methods:**

Using multivariable-adjusted cubic splines, we examined the associations of daily VILPA duration with overall MACE and its subtypes (incident myocardial infarction, heart failure and stroke) among non-exercisers (individuals self-reporting no leisure-time exercise and no more than one recreational walk per week) in the UK Biobank. We also undertook analogous analyses for vigorous physical activity among exercisers (individuals self-reporting participation in leisure-time exercise and/or recreational walking more than once a week).

**Results:**

Among 13 018 women and 9350 men, there were 331 and 488 all MACE, respectively, over a 7.9-year follow-up. In women, daily VILPA duration exhibited a near-linear dose–response association with all MACE, myocardial infarction and heart failure. In men, dose-reponse curves were less clear with less evidence of statistical signifigance. Compared with women with no VILPA, women’s median daily VILPA duration of 3.4 min was associated with hazard ratios (HRs; 95% confidence intervals) of 0.55 (0.41 to 0.75) for all MACE and 0.33 (0.18 to 0.59) for heart failure. Women’s minimum doses of 1.2–1.6 min of VILPA per day were associated with HRs of 0.70 (0.58 to 0.86) for all MACE, 0.67 (0.50 to 0.91) for myocardial infarction, and 0.60 (0.45 to 0.81) for heart failure. The equivalent analyses in UK Biobank’s accelerometry sub-study exercisers suggested no appreciable sex differences in dose–response.

**Conclusions:**

Among non-exercising women, small amounts of VILPA were associated with a substantially lower risk of all MACE, myocardial infarction and heart failure. VILPA may be a promising physical activity target for cardiovascular disease prevention, particularly in women unable or not willing to engage in formal exercise.

**WHAT IS ALREADY KNOWN ON THIS TOPIC**

- Higher levels of physical activity are associated with a reduced risk of experiencing major adverse cardiovascular events, such as cardiovascular disease-related death, myocardial infarction (heart attack), heart failure or stroke.
- Vigorous intermittent lifestyle physical activity (VILPA, intense physical activity accrued in very short bouts that is embedded into daily life) is beneficially associated with cardiovascular disease related mortality.
- There is a lack of evidence on how VILPA, and physical activity intensity in general, affect major cardiovascular events differently in men and women who do not exercise.

**WHAT THIS STUDY ADDS**

- In women, VILPA exhibited a near-linear dose–response association with most major adverse cardiovascular events. Such associations were less evident in men.
- Small amounts of VILPA in women were associated with substantially lower risk of overall major adverse cardiovascular events, myocardial infarction and heart failure.
- Women’s median VILPA duration of 3.4 min per day was associated with 45% (25% to 59%) lower risk of overall major adverse cardiovascular events; and with 67% (41% to 82%) lower risk of heart failure.

**HOW THIS STUDY MIGHT AFFECT RESEARCH, PRACTICE OR POLICY**

- VILPA may be a promising physical activity target for major cardiovascular events prevention in women unable or not willing to engage in formal exercise.
- In addition to regular VILPA, men may benefit from engaging in some structured exercise of vigorous intensity.

## Introduction

 Cardiovascular disease (CVD) is the leading cause of death in both men and women globally.^1^ Major adverse cardiovascular events (MACE), defined as non-fatal stroke/myocardial infarction/heart failure or cardiovascular death,^2^ are a commonly used composite of main CVD outcomes in trials and observational studies. Clinical and public health practice have traditionally focused on the cardio-protective properties of longer bouts of physical activity carried out during structured exercise sessions. The dose–response associations shorter bouts of vigorous intensity physical activity and cardiovascular outcomes is less clear. Compared with lower intensities, vigorous physical activity (VPA: ≥6 absolute metabolic equivalents of task—for example, stair climbing or running) elicits more pronounced cardiovascular effects^3^,^6^ in a shorter period of time.^7^ Despite these advantages, vigorous intensity exercise is not feasible or appealing to most middle-aged adults.^8^

High-intensity interval training (HIIT)^9^ studies have shown that, when repeated regularly, short bursts of vigorous intensity exercise can result in substantial improvements in cardiorespiratory fitness and other cardiovascular outcomes. Drawing on an analogous principle, vigorous intermittent lifestyle physical activity (VILPA)^4 10^ refers to brief and sporadic (eg, up to 1 min long)^4 11^ bouts that are done during daily living. Since short bouts of physical activity cannot be captured by questionnaires, wearable trackers are essential for measuring VILPA.^4 11^ A recent study in non-exercisers^4^ (ie, individuals reporting no leisure-time exercise) found a beneficial association of daily VILPA with cardiovascular mortality, although the relatively small number of fatal events precluded a detailed examination of dose–response, or examining sex-specific associations with CVD subtypes.

Sex differences in pathophysiology may moderate the influence of risk factors (including physical activity) on heart failure, myocardial infarction and stroke.^12^ Women have lower cardiorespiratory fitness on average than men at any given age,^13^ making the level of physical effort for a given physical task (and hence the physiological stimulus for adaptation) higher for women. Despite the established sex differences in fitness, and in vascular, muscular and respiratory responses to physical activity of higher intensity,^14^ there is no evidence as to whether sex differences exist in the long-term cardiovascular health effects of vigorous physical activity. Such evidence is critical to inform appropriate sex-specific clinical and public health guidelines for CVD prevention.^15^

The aim of this study was to examine the sex-specific dose–response associations of daily VILPA duration and frequency with MACE and its subtypes, and estimate minimal VILPA amounts for MACE risk reduction. To understand the role of the context in which physical activity takes place, we also examined in the same cohort the analogous dose–response associations of overall vigorous physical activity (coming from exercise or lifestyle physical activity) with the same MACE outcomes among exercisers.

## Methods

### Sample and design

The UK Biobank Study is a prospective cohort study of adults aged between 40 and 69 years at baseline (2006–2010). Participants provided informed consent, and ethical approval was provided by the UK’s National Health Service, National Research Ethics Service (Ref 80 11/NW/0382) study.

Between 2013 and 2015, 103 684 UK Biobank participants used a wrist-worn accelerometer for 7 days.^16^ ^17^ We defined a valid monitoring day as wear time greater than 16 hours. To be included in the analysis, participants were required to have at least three valid monitoring days, including at least 1 weekend day.^4 5 11 18^ We excluded participants with insufficient valid wear days, those who had missing covariate data and participants who reported an inability to walk. Online supplemental eFigure 1 shows the derivation of the core analytic samples of non-exercisers.

As previously described,^4 11^ we examined VILPA by separating UK Biobank accelerometry substudy participants who self-reported no leisure time exercise participation and no more than one recreational walk per week.^4 11^ To provide a comparison between the sex-specific effects of VILPA and (context-agnostic) vigorous physical activity, we repeated the main analyses among the accelerometry substudy participants who self-reported participation in any leisure-time exercise or more than one recreational walk per week^4^ (online supplemental eFigure 2).

### Physical activity assessment and exposure variables

We have described the physical activity intensity classification method in detail elsewhere^4 5 11^ and in online supplemental eText 1. In brief, physical activity intensity was classified into light, moderate and vigorous using a validated^4 5^ two-stage machine learning-based Random Forest activity classifier. In the 88 non-exercisers from the Australian validation sample, the correct classification of predicted VILPA against ground truth was >94.0% for both women and men (online supplemental eText 1– eText Tables 4a and 4b).

We considered short bouts lasting up to 2 min, based on a recent study^19^ among 70 middle-aged adults (58.0±9.6 years; 35 female) showing that the mean (SD) time required to verify physiological strain equating to vigorous intensity during typical VILPA activities is 76.7 (3.8) s. Considering that 96.2% of all VILPA bouts in our UK Biobank sample lasted up to 1 (89.1%) or over 2 (7.1%) min, and only 3.8% of bouts lasted 1–2 min, we present detailed data for bouts up to 1 min with only some indicative results presented for bouts lasting up to 2 min. As previously described,^4^ for daily VILPA frequency analyses we length-standardised bouts to 1 min, for comparability with previous work^4^ and a more concrete interpretation of the corresponding effect sizes (online supplemental eText 2). For example, the length of raw bouts varied from 10 to 60 s, length-standardising each bout to 1 min permits an interpretation of the daily VILPA frequency results that is not conditional on the length of each bout. For completeness, we also present VILPA frequency analyses with raw (unstandardised) bouts. Considering that our main exposure refers to a range of duration (ie, bouts lasting from 10 to 60 s), the advantage of the effect sizes of the length-standardised bouts is that they refer to a specific dose of VILPA, contrary to unstandardised bouts, which reflect average bout duration.

We have previously^4 11^ described in detail the selection of a sample of non-exercisers. We used information on participation in leisure-time exercise and recreational walking available in the 2006–2010 baseline of the UK Biobank study. Among the 6095 UK Biobank accelerometry sample participants who self-reported no exercise at the baseline and had a re-examination on average 1.5 years (SD 1.4) prior to the 2013–2015 accelerometry substudy, 88% maintained their non-exercise status over time.

### Mortality and MACE ascertainment

Participants were followed up through 30 November 2022, with deaths obtained via linkage with the National Health Service (NHS) Digital of England and Wales or the NHS Central Register and National Records of Scotland. Inpatient hospitalisation data were provided by either the Hospital Episode Statistics for England, the Patient Episode Database for Wales, or the Scottish Morbidity Record for Scotland. MACE was defined as death or incidence of ST-elevated or non-ST elevated myocardial infarction (International Classification of Diseases version 10: I21, I23, I24, I25, I26, I30, I31, I33, I34, I35, I38, I42, I45, I46, I48), stroke (I60, I61, I63, I64, I67), and heart failure (I11, II13, I50, I51).

### Statistical analyses

To reduce the risk of reverse causation through prodromal/undiagnosed disease, we excluded those with an event within the first 2 years of follow-up and those with prevalent CVD at the accelerometry baseline. The upper range of VILPA/VPA values were winsorised at the 97.5 percentile to minimise the effect of sparse data or outliers.^4 5 11^

Using Fine-Gray subdistribution hazards to account for competing risks from non-CVD deaths,^20^ we examined dose–response of average daily duration, and length-standardised and raw frequency of VILPA bouts, as well as the corresponding (context-agnostic) VPA variables in exercisers. Since the distribution of primary exposures (VILPA and VPA) were highly skewed, knots of the restricted cubic splines were placed on the higher-density data areas^21^ at equally distributed frequencies (10th, 33rd, 67th percentiles). Departure from linearity was assessed by a Wald test. Proportional hazards assumptions were tested using Schoenfeld residuals in the models with all three outcomes, and no violations were observed (all p>0.05). We tested for potential non-linearity using Wald tests. Based on relevant previous observational vigorous physical activity literature^4 5 11^ and a analysis-specific directed acyclic graph (see online supplemental eText 5), we selected the following covariates to adjust the core models (see supplemental eTable 7): age, average daily duration of light and moderate intensity physical activity, average duration of VILPA/VPA coming from bouts lasting over 1 or 2 min, smoking history, alcohol consumption, accelerometer-estimated sleep duration, diet, education, ethnicity, self-reported parental history of CVD, prevalent cancer and self-reported medication use (cholesterol, blood pressur, and diabetes). To prevent multicollinearity, the raw frequency of VILPA was adjusted for the residual of VILPA duration. Interaction effects by sex were assessed and decisions to plot interaction versus independent (sex-stratified) models were informed by these findings (online supplemental eTable 9). The reference data point for all main models were zero minutes of VILPA/VPA per day.^4^ Analyses for each outcomes employed a slightly different sample size owing to outcome-specific exclusions of prevalent disease.

To provide conservative point estimates we calculated the ‘minimal dose’, defined as VILPA/VPA, volume/frequency associated with 50% of the optimal risk reduction.^4 5 22 23^ We also present point estimates (hazard ratios and 95% confidence intervals) associated with the median volume/frequency VILPA/VPA values. We calculated E-values to estimate the plausibility of bias from unmeasured confounding. To provide a broader physical activity context to sex differences, we also examined the dose–response curves of light and moderate intensity physical activity against MACE outcomes in non-exercisers.

We conducted sensitivity analyses of VILPA with additional adjustment for potential mediators—namely, glycated haemoglobin (HbA1c), low-density lipoprotein, high-density lipoprotein, triglycerides, systolic blood pressure, diastolic blood pressure, and body mass index. To further reduce reverse causation bias, we also excluded participants who had poor self-rated health or a body mass index below 18.5 kg/m^2^ or current smokers^4^ or a frailty index ≥3 (on a 0 to 5 scale).^24^ To assess the influence of variations of the reference data point on estimates, we repeated sex-specific analyses of VILPA duration using the 15th percentile of the VILPA duration distribution as referent (0.63 min of VILPA per day). We further tested robustness of the results by running a sensitivity analysis, where we replaced Fine-Gray distribution hazards with cause-specific hazard models.^25^ To assist the interpretation of our findings we calculated physical activity energy expenditure during VILPA bouts and relative physical activity intensity (%VO_2_max) during VILPA bouts in the subsample of 2043 women and 1588 men non-exercisers with valid accelerometry and fitness test data (online supplemental eText 3). To examine the influence of relative intensity as an explanation of any observed differences in the dose–response of VILPA with MACE outcomes, we ran a set of sensitivity analyses where we defined vigorous intensity as >7 and >8 metabolic equivalents (MET).

We performed all analyses using R statistical software (version 4.2.3) with the RMS (version 6.3.0) and survival packages (version 3.5.5). We reported this study according to Strengthening the Reporting of Observational Studies in Epidemiology (STROBE) guidelines (online supplemental eTable 10) and the CHecklist for statistical Assessment of Medical Papers (CHAMP) statement.^26^

### Patient and public involvement

This study did not involve patients or members of the public in the planning, design, data collection, analysis or interpretation of results.

### Equity, diversity and inclusion statement

Our study sample represents all participants who took part in the UK Biobank study and provided valid accelerometer data, reflecting the demographic, geographical and socioeconomic diversity of the participants.

## Results

Sample online supplemental eFigure 1 shows the sample derivation process which resulted in 22 368 (13 018 women/9350 men) UK Biobank participants being included in the analyses, corresponding to 819 MACE events (331 female/488 male). Slightly smaller sample sizes were entered into the analysis of myocardial infarction (n=21 928; 379 events (129 female/250 male)), heart failure (n=21 764; 215 events (96 female/119 male)), and stroke (n=21 774; 225 events (106 female/119 male)). We also considered analyses for stroke subtypes, but these were less feasible due to the very low number of events for haemorrhagic stroke (n=21 596; 47 events (30 female/17 male)) vs ischaemic (n=21 774; 175 events (76 female/99 male)). The censoring proportion in our study was 93.75%, with participants censored due to the absence of an event until follow-up in November 2022.

Table 1 presents the characteristics of the sample by sex and daily VILPA duration. The mean age of participants was 61.9 (7.6) years and mean follow-up was 7.9 (1.0) years corresponding to 176 678 person-years. To understand the role of how confounding by indication might have influenced the sex-specific findings, online supplemental eText 4 provides a comparison between the referent groups of women and men. Online supplemental eFigure 2**,** online supplemental eTable 1**,** and online supplemental eText 4 describe the characteristics of the exercisers sample. Online supplemental eTable 2 presents details of bout length of VILPA (non-exercisers) and VPA (exercisers).

**Table 1:** Participant baseline characteristics by VILPA duration and sex (non-exercisers, n= 22 368)

	Female				Male			
Tertiles of VILPA duration (min/day)	0	0.1–2.5	2.5–7.5	7.5	0	0.1–2.5	2.5–7.5	7.5
Sample size (n)	969	4017	4016	4016	371	2993	2993	2993
Follow-up (in years), mean (SD)	7.8 (1.2)	7.9 (1.0)	8.0 (0.9)	8.0 (0.8)	7.5 (1.5)	7.8 (1.2)	7.9 (1.1)	7.9 (1.0)
Age (in years), mean (SD)	64.8 (6.8)	62.8 (7.5)	60.9 (7.5)	59.6 (7.4)	65.7 (6.6)	64.1 (7.3)	62.4 (7.6)	60.6 (7.8)
Ethnicity - white, n (%)	937 (96.7)	3878 (96.5)	3850 (95.9)	3819 (95.1)	365 (98.4)	2896 (96.8)	2891 (96.6)	2848 (95.2)
Smoking history, n (%)								
Current	112 (11.6)	347 (8.6)	307 (7.6)	265 (6.6)	72 (19.4)	340 (11.4)	313 (10.5)	274 (9.2)
Never	538 (55.5)	2355 (58.6)	2487 (61.9)	2510 (62.5)	154 (41.5)	1455 (48.6)	1554 (51.9)	1631 (54.5)
Previous	319 (32.9)	1315 (32.7)	1222 (30.4)	1241 (30.9)	145 (39.1)	1198 (40.0)	1126 (37.6)	1088 (36.4)
Alcohol consumption, n (%)*								
Never	48 (5.0)	220 (5.5)	163 (4.1)	185 (4.6)	11 (3.0)	76 (2.5)	70 (2.3)	71 (2.4)
Ex-drinker	45 (4.6)	159 (4.0)	145 (3.6)	105 (2.6)	12 (3.2)	107 (3.6)	70 (2.3)	89 (3.0)
Within guidelines	667 (68.8)	2749 (68.4)	2779 (69.2)	2751 (68.5)	187 (50.4)	1417 (47.3)	1394 (46.6)	1455 (48.6)
Above guidelines	209 (21.6)	889 (22.1)	929 (23.1)	975 (24.3)	161 (43.4)	1393 (46.5)	1459 (48.7)	1378 (46.0)
Education, n (%)								
College	336 (34.7)	1411 (35.1)	1443 (35.9)	1493 (37.2)	159 (42.9)	1215 (40.6)	1140 (38.1)	1111 (37.1)
A/AS level	135 (13.9)	531 (13.2)	554 (13.8)	550 (13.7)	47 (12.7)	324 (10.8)	349 (11.7)	349 (11.7)
O level	223 (23.0)	969 (24.1)	973 (24.2)	968 (24.1)	60 (16.2)	522 (17.4)	602 (20.1)	570 (19.0)
CSE	36 (3.7)	196 (4.9)	214 (5.3)	249 (6.2)	9 (2.4)	125 (4.2)	138 (4.6)	206 (6.9)
NVQ/HND/HNC	36 (3.7)	152 (3.8)	142 (3.5)	131 (3.3)	27 (7.3)	273 (9.1)	280 (9.4)	304 (10.2)
Other	203 (20.9)	758 (18.9)	690 (17.2)	625 (15.6)	69 (18.6)	534 (17.8)	484 (16.2)	453 (15.1)
Fruit and vegetable consumption, n (%)†								
Low	404 (41.7)	1695 (42.2)	1677 (41.8)	1646 (41.0)	158 (42.6)	1240 (41.4)	1223 (40.9)	1193 (39.9)
Moderate	310 (32.0)	1298 (32.3)	1296 (32.3)	1352 (33.7)	97 (26.1)	777 (26.0)	788 (26.3)	770 (25.7)
High	255 (26.3)	1024 (25.5)	1043 (26.0)	1018 (25.3)	116 (31.3)	976 (32.6)	982 (32.8)	1030 (34.4)
Medication, n (%)								
Cholesterol	184 (19.0)	493 (12.3)	369 (9.2)	239 (6.0)	102 (27.5)	697 (23.3)	541 (18.1)	410 (13.7)
Insulin	15 (1.5)	27 (0.7)	22 (0.5)	17 (0.4)	7 (1.9)	33 (1.1)	23 (0.8)	16 (0.5)
Blood pressure	263 (27.1)	734 (18.3)	526 (13.1)	377 (9.4)	114 (30.7)	759 (25.4)	634 (21.2)	420 (14.0)
Diagnosed cancer, n (%)	147 (15.2)	552 (13.7)	439 (10.9)	381 (9.5)	28 (7.5)	249 (8.3)	204 (6.8)	150 (5.0)
Family history of CVD, n (%)	591 (61.0)	2330 (58.0)	2230 (55.5)	2162 (53.8)	182 (49.1)	1569 (52.4)	1573 (52.6)	1480 (49.4)
Light activity (min/day), median (IQR)	81.2 (83.6)	86.5 (82.7)	86.1 (73.9)	88.2 (70.5)	77.9 (71.5)	88.4 (80)	88.9 (78.6)	93.4 (71.4)
Moderate activity (min/day), median (IQR)	10.8 (14.5)	17.9 (20.2)	25.2 (23.8)	36.7 (30.5)	8.8 (10.8)	15.7 (17.2)	23.4 (21.8)	33.3 (28.9)
Sleep duration (min/day), median (IQR)	438.5 (84)	439 (78.5)	443.5 (74.6)	441.1 (71.3)	426.7 (89.3)	433.5 (83.2)	434.2 (78.8)	432.8 (77.6)
Glycated haemoglobin (HbA1c), mean (SD)	37.3 (7.9)	36.0 (5.8)	35.2 (4.7)	34.8 (4.6)	38.9 (9.6)	37.1 (8.6)	35.9 (6.4)	35.3 (5.4)
HDL (mmol/L), mean (SD)	1.5 (0.4)	1.5 (0.4)	1.6 (0.4)	1.6 (0.4)	1.2 (0.3)	1.2 (0.3)	1.3 (0.3)	1.3 (0.3)
LDL (mmol/L), mean (SD)	3.7 (0.9)	3.7 (0.9)	3.7 (0.8)	3.6 (0.8)	3.4 (0.9)	3.5 (0.9)	3.6 (0.8)	3.6 (0.8)
Triglycerides (mmol/L), mean (SD)	1.7 (0.9)	1.7 (0.9)	1.6 (0.8)	1.4 (0.8)	2.2 (1.2)	2.1 (1.2)	2.1 (1.2)	2.0 (1.1)
Diastolic blood pressure (mm Hg), mean (SD)	82.6 (10.2)	81.9 (10.5)	80.8 (10.4)	79.5 (10.1)	85.1 (10.5)	85.4 (10.4)	84.7 (10.3)	83.8 (10.4)
Systolic blood pressure (mm Hg), mean (SD)	140.8 (19.7)	138.4 (19.5)	136.0 (19.2)	133.9 (19.0)	145.3 (17.8)	144.3 (17.9)	142.6 (17.5)	141.1 (17.5)
Body mass index (kg/m^2^), mean (SD)	29.3 (5.9)	28.3 (5.8)	27.0 (5.2)	25.6 (4.5)	29.6 (5.5)	28.6 (4.6)	27.7 (4.3)	26.9 (3.9)
MACE incidence, n (%)	52 (5.4)	141 (3.5)	80 (2.0)	58 (1.4)	34 (9.2)	189 (6.3)	150 (5.0)	115 (3.8)
MACE subtype								
Myocardial infarction	18 (1.8)	60 (1.5)	29 (1)	22 (0.7)	10 (2.7)	101 (3.3)	76 (2.5)	63 (2.1)
Heart failure	24 (2.5)	40 (0.9)	22 (1)	10 (0.5)	16 (4.3)	41 (1.4)	38 (1.3)	24 (0.8)
Stroke‡	10 (1.0)	41 (1.0)	30 (1)	25 (0.6)	8 (2.2)	50 (1.7)	34 (1.1)	27 (0.9)
Haemorrhagic	1 (0.1)	8 (0.2)	12 (0.3)	9 (0.2)	0 (0)	6 (0.2)	6 (0.2)	5 (0.2)
Ischaemic	9 (0.9)	33 (0.8)	18 (0.5)	16 (0.4)	6 (1.6)	43 (1.4)	28 (0.9)	22 (0.7)

The columns breakdown corresponds to *d*uration of VILPA bouts. Values represent mean (SD) unless specified otherwise.

* Alcohol consumption: above guidelines are >14 units per week, where 1 unit = 8 g of ethanol.

† Fruits and vegetable consumption: low is <5 servings per day, high is >8 servings per day.

‡ There were three cases in stroke which was undetermined. Thus, they do not add to total stroke incidence. IQR: interquartile range.

CVDcardiovascular diseaseHDLhigh-density lipoproteinLDLlow-density lipoproteinMACEmajor adverse cardiovascular events VILPAvigorous intermittent lifestyle physical activity

### Dose–response associations of VILPA with MACE and its subtypes

**Figure 1:**
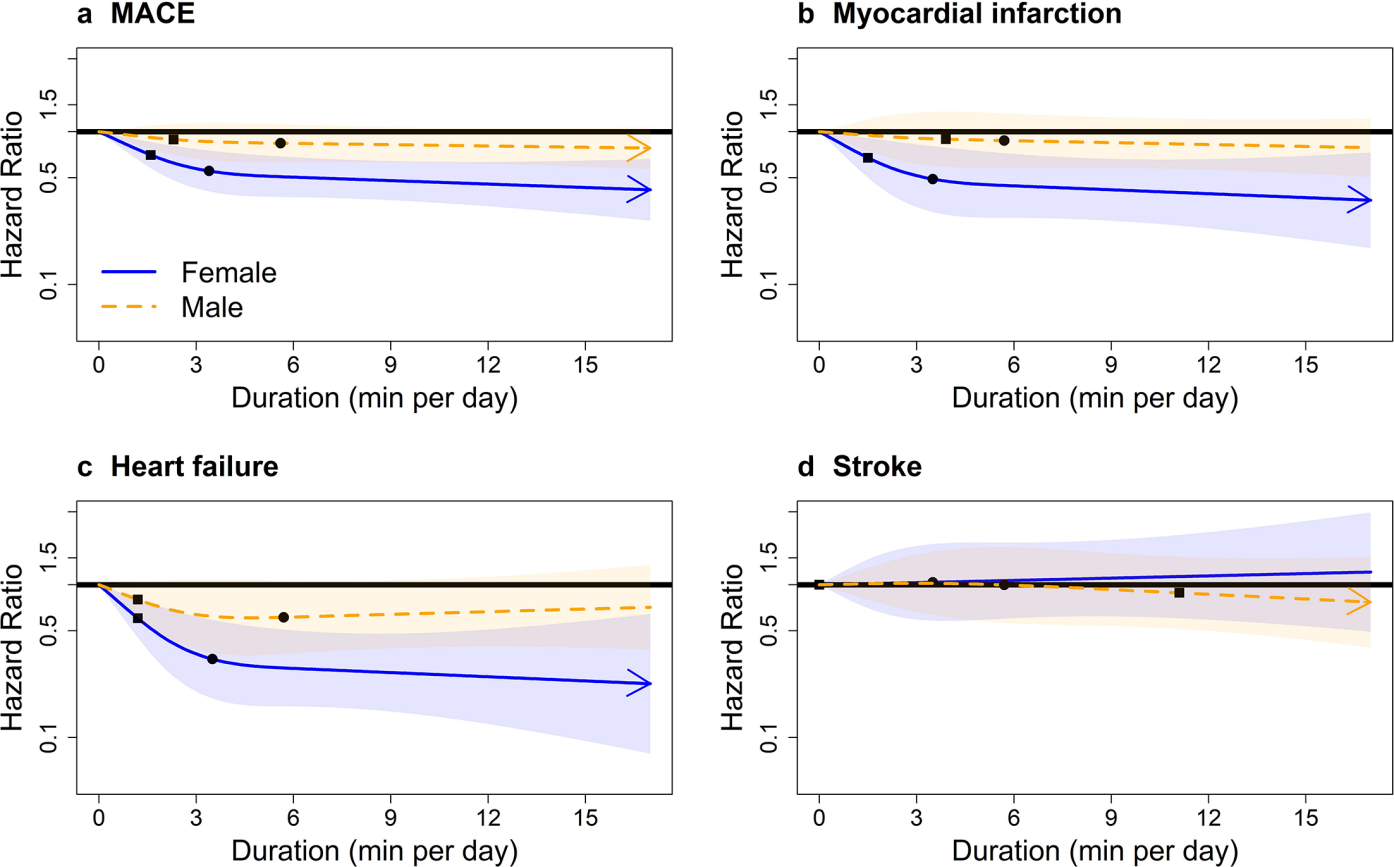
Sex-specific adjusted dose–response curves of the duration of daily vigorous intermittent lifestyle physical activity (VILPA) for major adverse cardiovascular events (MACE) and its subtypes, bouts lasting up to 1 min (min/day). Adjusted for age, light intensity, moderate intensity, VILPA bouts over 1 min, smoking history, alcohol consumption, accelerometer-estimated sleep duration, diet, education, ethnicity, self-reported parental history of cardiovascular disease, previous incidence of cancer and self-reported medication use (for cholesterol, blood pressure and diabetes). Bold lines represent HRs, while shaded areas indicate 95% CI. (A) All MACE: n=22 368; events: 819 (female/male=331/488). (B) Myocardial infarction: n=21 928; events=379 (female/male=129/250). (C) Heart failure: n=21 764; events=215 (female/male=96/119). (D) Stroke: n=21 774; events=225 (female/male=106/119). Squares, minimal dose, as indicated by the ED_50_ statistic, which estimates the daily duration of VILPA associated with 50% of optimal risk reduction. Circles, HR associated with the median VILPA value (see online supplemental eTable 3 for the list of values).

Multivariable-adjusted absolute risk dose–response curves of VILPA for MACE and subtypes by sex are presented in online supplemental eFigure 3. The relative risk analyses showed clear dose–response associations only in women for total MACE, myocardial infarction, and heart failure (figure 1). For example, for MACE the median daily VILPA duration doses (3.4/5.6 min for women/men) were associated with HRs of 0.55 (0.41 to 0.75) in women and 0.84 (0.63 to 1.12) in men; for heart failure the HRs were 0.33 (0.18 to 0.59) in women and 0.61 (0.35 to 1.06) in men (online supplemental eTable 3). We found statistically significant multiplicative sex*VILPA interactions for MACE, myocardial infarction and heart failure, but not for stroke. We also found additive interactions^27^ of sex*VILPA for MACE, heart failure and stroke (online supplemental eTable 9).

**Figure 2:**
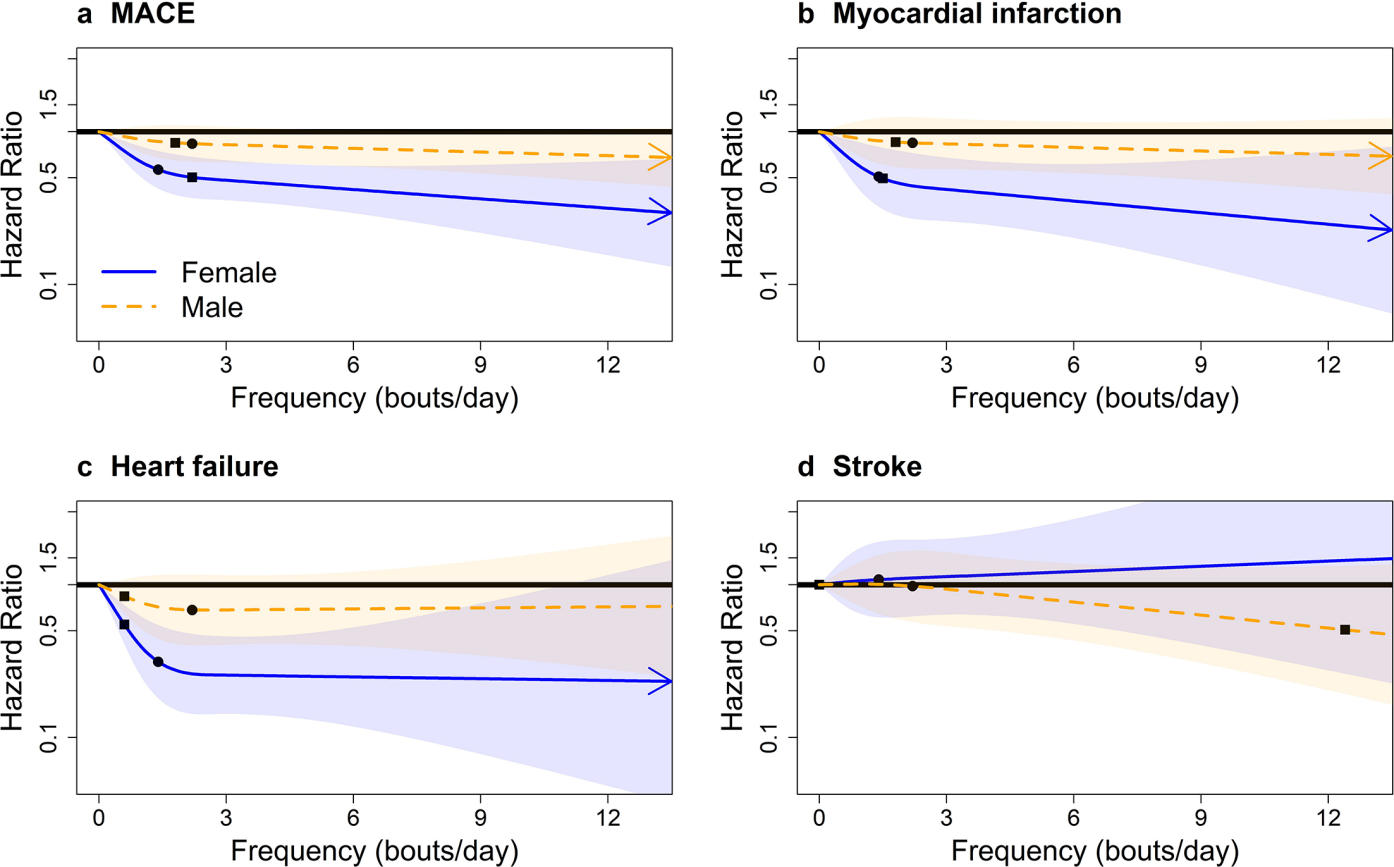
Sex-specific adjusted dose–response curves for major adverse cardiovascular events (MACE) and its subtypes by length-standardised vigorous intermittent lifestyle physical activity (VILPA) frequency; bouts lasting up to 1 min (bouts/day). Adjusted for age, light intensity, moderate intensity, VILPA bouts over 1 min, smoking history, alcohol consumption, accelerometer-estimated sleep duration, diet, education, ethnicity, self-reported parental history of cardiovascular disease, previous incidence of cancer and self-reported medication use (for cholesterol, blood pressure and diabetes). The range was capped at the 97.5 percentile to minimise the influence of sparse data. Bold lines represent HRs, while shaded areas indicate 95% CI. (A) All MACE: n=22 368; events: 819. (B) Myocardial infarction: n=21 928; events=379. (C) Heart failure: n=21 764; events=215. (D) Stroke: n=21 774; events=225. Squares, minimal dose, as indicated by the ED_50_ statistic, which estimates the daily duration of VILPA associated with 50% of optimal risk reduction. Circles, HR associated with the median VILPA value (see online supplemental eTable 3 for the list of values).

Length-standardised and raw daily VILPA frequency showed similar dose–response patterns with VILPA duration across all outcomes in both sex groups (figure 2, online supplemental eFigure 4). The median VILPA length-standardised bouts frequency (1.4/2.2 bouts per day in women/men) was associated with HRs of 0.56 (95% CI 0.42 to 0.75) in women and 0.83 (95% CI 0.60 to 1.10) in men for MACE; and HRs of 0.31 (95% CI 0.18 to 0.54) in women and 0.68 (95% CI 0.40 to 1.16) in men for heart failure. For MACE in women, the median raw frequency dose of 9.3 bouts per day was associated with a HR of 0.63 (95% CI 0.46 to 0.87). For MACE in men, the median raw frequency dose of 11.4 raw bouts per day was associated with a HR of 0.76 (95% CI 0.56 to 1.02) (online supplemental eTable 3). Men’s median raw frequency was associated with lower risk of heart failure (HR of 0.49, 95% CI: 0.28 to 0.87, respectively). Dose–response analyses of daily duration for VILPA bouts lasting up to 2 min elicited very similar results to bouts lasting up to 1 min (online supplemental eFigure 5).

Minimum daily doses: online supplemental eTable 3 presents the HR and 95%confidence interval associated with the minimum dose (eliciting 50% of the total effect),^4 5 22 23^ for VILPA bouts lasting up to 1 min. For MACE in women and men, the minimum duration dose was 1.6 and 2.3 min per day, corresponding to HRs of 0.70 (95% CI 0.58 to 0.86) and 0.89 (95% CI 0.70 to 1.12), respectively. Findings were analogous for the minimum doses of myocardial infarction (1.5/3.9 min per day for women/men) and heart failure (1.2 min per day in both sex groups), which were statistically significant for women only (HR=0.67 (95% CI 0.50 to 0.91) and 0.60 (95% CI 0.45 to 0.81), respectively) (online supplemental eTable 3).

For overall MACE in women, the minimum frequency dose was 2.2 length-standardised bouts and 9.6 raw bouts per day corresponding to HRs of 0.50 (95% CI 0.37 to 0.68) and 0.63 (95% CI 0.46 to 0.86), respectively. For overall MACE in men, the minimum frequency dose was 1.7 length-standardised bouts and 4.4 raw bouts per day corresponding to HRs of 0.85 (95% CI 0.65 to 1.10) and 0.86 (95% CI 0.71 to 1.03), respectively. For heart failure, men’s minimum dose was 3.1 bouts per day, corresponding to a statistically significant HR of 0.74 (95% CI: 0.58 to 0.95) (online supplemental eTable 3).

### Comparisons of dose–response associations in exercisers and non-exercisers

**Figure 3:**
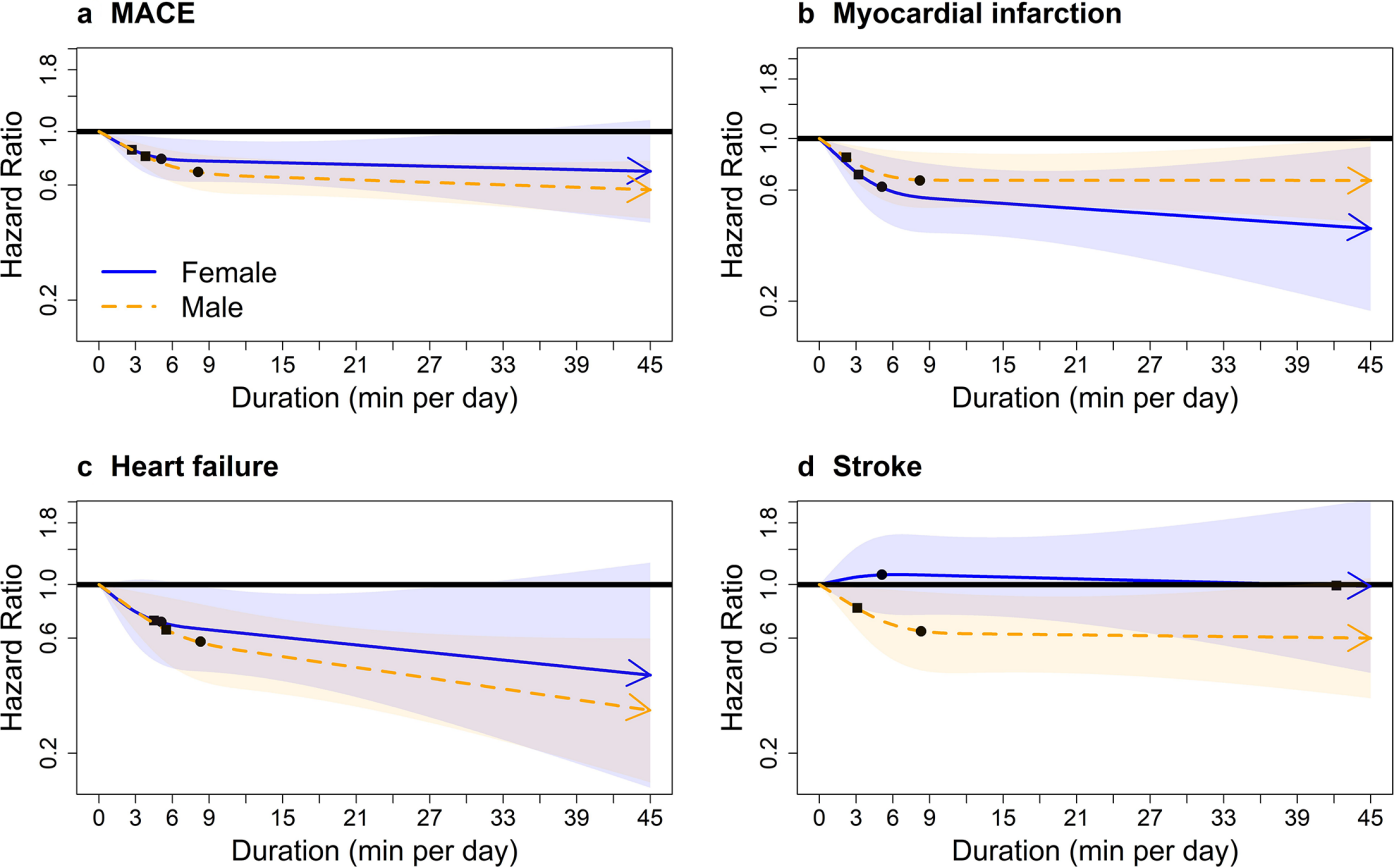
Adjusted sex-specific dose–response curves of vigorous physical activity (VPA) in exercisers for major adverse cardiovascular events (MACE) and its subtypes, bouts lasting up to 1 min (min/day). Adjusted for sex, age, light intensity, moderate intensity, VPA bouts over 1 min, smoking history, alcohol consumption, accelerometer-estimated sleep duration, diet, education, ethnicity, self-reported parental history of cardiovascular disease, previous incidence of cancer and self-reported medication use (for cholesterol, blood pressure and diabetes). The range was capped at the 97.5 percentile to minimise the influence of sparse data. Bold lines represent HRs, while shaded areas indicate 95% CI. (A) MACE: n=58 648; events=1854 (female/male=749/1105). (B) Myocardial infarction: n=57 622; events=828 (female/male=287/541). (C): Heart failure: n=57 289; events=495 (female/male=210/285). D) Stroke: n=57 325; events=531 (female/male=252/279). Squares, minimal dose, as indicated by the ED_50_ statistic, which estimates the daily duration of VPA associated with 50% of optimal risk reduction. Circles, HR associated with the median VPA value (see online supplemental eTable 4 for the list of values).

The range of average daily VPA in exercisers was considerably wider than that of VILPA in non-exercisers (0–45 vs 0–17 min per day), as well as the bout length (online supplemental eTable 2). Among exercisers there were no major sex differences in the dose–response associations of VPA with overall MACE, myocardial infarction or heart failure, while there was evidence of a dose–response association with stroke only in men (figure 3). For MACE, male exercisers’ median daily VPA duration value of 8.1 min was associated with a HR of 0.68 (95% CI 0.57 to 0.80) (online supplemental eTable 4). The results of the length-standardised (online supplemental eFigure 6) and raw (online supplemental eFigure 7) daily VPA frequency in exercisers were broadly consistent with the equivalent VPA duration findings.

### Sensitivity and additional analyses

All sensitivity analyses produced results consistent with the main findings ([Supplementary-material SP1] online supplemental eFigure 8-11/15-17 and online supplemental eText 4). For example, repeating analyses using cause-specific hazard models had minimal impact on the dose-response curves (online supplemental eFigure 15-17). Analyses with the alternative MET definitions of VILPA (non-exercisers) or VPA (exercisers) showed that the shift to 7 or 8 MET as a vigorous intensity threshold strengthened the dose–response associations of women’s VILPA with overall MACE, myocardial infarction, stroke (8 MET threshold only) and heart failure; but had no material influence on men’s VILPA curves (online supplemental eFigure 18-19). In exercisers, the alternative MET thresholds produced results consistent with the main analyses using the conventional 6 MET threshold (online supplemental eFigure 20-21).

In the older age group (>62.4 years, n=11 673; 618 MACE events), the dose–response associations of VILPA with MACE, myocardial infarction and heart failure closely mirrored the sex-specific patterns in the whole sample of non-exercisers (online supplemental eFigure 12). In the younger age group (n=10 695), associations were less clear, possibly owing to the small number of events. The age*VILPA multiplicative interaction tests were not statistically significant (online supplemental eTable 8), possibly for the same reason. With the exception of light intensity and heart failure, we noted no other major sex differences in the dose–response of light and moderate intensity physical activity with MACE and its subtypes in non-exercisers (online supplemental eFigure 13-14). E-values indicated that for our estimates in women to be null, the association of an unmeasured confounder with VILPA duration exposures and outcomes should be a HR (lower 95% CI) of 2.21 (1.60) to 3.04 (2.00) for MACE; 2.34 (1.42) to 3.49 (1.80) for myocardial infarction; or 2.72 (1.77) to 5.51 (2.78) for heart failure. In men, for our estimates to be null, the association of an unmeasured confounder should be a HR (lower 95% CI) of 1.81 (1.00)–2.66 (1.00) for heart failure (online supplemental eTable 5). Analogous e-values for exerciusers are presented in online supplemental eTable 6. In the subsample of 2043 female and 1588 male non-exercisers with valid accelerometry and fitness data (online supplemental eText 3), the average absolute VO_2_ during VILPA bouts was 6.04 (1.02) MET for women and 6.21 (1.52) MET for men, corresponding to a relative intensity of 83.2 (18.2)% of VO_2_max for women and 70.5 (22.1)% of VO_2_max for men (online supplemental eTable 11).

## Discussion

Current clinical and public health guidelines assume similar cardiovascular responses to physical activity between sexes and offer no guidance on what quantity of incidental (non-exercise) activity has benefit. Our study, which uniquely examined sex differences in the dose–response of incidental physical activity quantified by wearable trackers with major cardiovascular events, demonstrated a significant and linear dose–response association of VILPA with all MACE, myocardial infarction, and heart failure among women, but evidence of such associations was less clear in men. Women’s daily median VILPA duration of 3.4 min was associated with 45% lower hazards for MACE: 51% for myocardial infarction, and 67% for heart failure. Minimum doses of an average of around 1.5 VILPA min per day (range 1.2–1.6 min) were associated with 30%, 33%, and 40% lower hazards for all MACE, myocardial infarction and heart failure, respectively, for women.

HIIT^9^ and proof-of-concept studies of intermittent stair climbing^7^ have shown bursts of VPA as brief as 20 s to a few minutes in length, performed three to five times a day, can result in improvements in cardiorespiratory fitness in previously inactive young adults, providing a potential physiological basis^28^ for our findings. However, the observed sex differences remain unexplored in current literature owing to the under-representation of women in HIIT trials.^29^ Considering that accelerometers record absolute intensity, it is likely that women’s VILPA bouts reflect higher relative loads compared with men, which might lead to more pronounced physiological adaptations in the long term. The metabolic, contractile and haemodynamic properties of skeletal muscle differ between men and women, possibly moderating the response to the same absolute dose of vigorous exercise activities like VILPA.^14^ However, these explanations received mixed support by our sensitivity and additional analyses. For example, in our subgroup analyses (online supplemental eText 3) women had 26% lower VO2max than men, consistent with previous literature.^28^

Despite the very similar absolute energy expenditure of VILPA bouts between women and men (6.04–6.21 MET), the relative intensity during VILPA was indeed substantially higher in women (83.2% (women) vs 70.5% (men)) (online supplemental eTable 11). Such relative intensity would categorise women’s but not men’s VILPA exertion as high intensity, according to current HIIT protocols.^30 31^ The absence of major sex differences in the dose–response of light and moderate intensity physical activity of non-exercisers (online supplemental eFigure 13–14) also supports this interpretation as the overall level of exertion in these intensities is relatively modest and unlikely to elicit substantially different physiological responses between sex groups. On the other hand, if the higher relative effort required by women for a given activity in daily living explained the sex differences in VILPA dose–response, we would expect that shifting the absolute intensity VILPA threshold to above 6 MET in men would result in some convergence of men’s curves to resemble women’s beneficial dose–response. However, our sensitivity analyses presented in online supplemental eFigure 18–19, where we increased the absolute intensity VILPA thresholds to 7 and 8 MET, do not lend full direct support to this explanation. While in women a shift of the MET thresholds linearised the curves and strengthened the associations of VILPA with MACE, suggesting that relative intensity does play a role, there were no material changes in men’s curves. Therefore, it remains less clear if relative intensity is the primary explanation of the sex differences we reported.

The sex-specific effects we observed were restricted to non-exercisers, suggesting a likely moderating role of the context in which vigorous physical activity is performed and bout characteristics. For example, vigorous bouts (online supplemental eTable 2) were approximately 30% longer for exercisers than non-exercisers, a pattern that probably reflects the voluntary and sustained effort involved in leisure-time exercise activities. One possible explanation of the more consistent dose–response in female and male exercisers is that their vigorous bouts were longer and more likely to occur during activities designed for recreation and fitness. On the other hand, VILPA in non-exercisers is more likely to be functional, opportunistic and less voluntary (eg, occupation, housework or transportation).

### Strengths and limitations

We used device-based physical activity measurement and a validated^4^,^6^ two-stage machine learning-based intensity classifier. We cannot rule out the possibility of reverse causation bias, an inherent selection bias in HRs,^32^ time-varying nature of exposure,^33^ potential feedback between exposure and confounders, and residual confounding. However, our results were robust to comprehensive sensitivity analyses and the E-values indicated that unmeasured confounding is unlikely to fully explain the observed associations.

Our results were robust to different handling of outcome events^25^ as Fine-Gray and cause-specific hazard models produced very consistent results. Some VILPA activities might not be fully captured by accelerometers (eg, the extra physiological effort required from carrying a backpack would not be registered), although such measurement error probably leads to underestimation of the ‘true’ associations with MACE due to regression dilution bias.^34^

Although no formal validation study on the leisure time questionnaire we used to separate exercisers from non-exercisers exists, our previous work^4^ and the data we present in this manuscript (online supplemental eTable 2) support its convergent validity by, for example, demonstrating that vigorous bouts were considerably longer in exercisers than in non-exercisers. There was a median lag of 5.5 years between the UK Biobank baseline when covariates and leisure-time physical activity measurements were taken and the accelerometry study. However, covariates have been shown to be stable over time^35^ and non-exerciser status a stable factor over time (82–88% stability).^4^ Accelerometry-measured physical activity is generally stable over time in adults (eg, >90% of classification accuracy within one quartile over a period of 2–3 years).^36^ Although the UK Biobank had a low response rate (5.5%), our recent empirical work has shown that poor cohort representativeness does not materially influence the associations between physical activity and cardiovascular mortality in the UK Biobank.^37^

### Conclusions

Non-exercise vigorous incidental physical activity showed a beneficial dose–response with MACE outcomes, which was pronounced in women, among whom very small amounts of VILPA (eg, approximately 1.5 to 4 min per day) were associated with substantially lower risks of overall MACE, myocardial infarction and heart failure. Although these findings are observational, they suggest that VILPA may be promising physical activity target for CVD prevention among non-exercising women. The less pronounced VILPA associations in men suggest that for optimal cardioprotective benefits some exercise-based vigorous intensity physical activity would also be desirable. Our results support sex-specific physical activity guidelines for CVD prevention.^15^ Our approach shows that wearable devices combined with machine learning-based methods can reveal novel physical activity targets for CVD prevention, and important sex differences to guide future preventive practices and interventions.

## supplementary material

10.1136/bjsports-2024-108484 online supplemental file 1

## Data Availability

The data that support the findings of this study are available from the UK Biobank, but restrictions apply to the availability of these data, which were used under license for the current study, and so are not publicly available. Data are, however, available from the authors upon reasonable request and with permission of the UK Biobank. ES and MA had full access to all the data in the study and takes responsibility for the integrity of the data and the accuracy of the data analysis.
