# Supplementary material for: Device-measured vigorous intermittent lifestyle physical activity (VILPA) and major adverse cardiovascular events: evidence of sex differences
Source: Br J Sports Med. 2024 Oct 28;59(5):e108484. doi: 10.1136/bjsports-2024-108484 (PMC11874358; doi:10.1136/bjsports-2024-108484)
Supplement: online supplemental file 1 [file bjsports-59-5-s001.pdf]

Supplemental material

Device-measured vigorous intermittent lifestyle physical activity and major adverse cardiovascular events: evidence of sex differences

eTable 1: Exercisers' baseline characteristics by VILPA duration and sex (n= 58,648)

	Duration of VPA							
	Female				Male			
**Tertiles of VILPA duration (min/day)**	**0**	**0.1-2.5**	**≥2.5-7.5**	**≥7.5**	**0**	**0.1-2.5**	**≥2.5-7.5**	**≥7.5**
**Sample size (n)**	1,628	10,912	10,912	10,912	523	7,921	7,920	7,920
**Follow up (in years), mean (SD)**	7.9 (1.1)	8.0 (0.9)	8.0 (0.8)	8.0 (0.8)	7.7 (1.3)	7.8 (1.2)	7.9 (1.0)	8.0 (1.0)
**Age (in years), mean (SD)**	65.3 (7.1)	62.9 (7.5)	60.7 (7.6)	58.5 (7.5)	66.4 (6.8)	63.7 (7.7)	61.5 (8.0)	59.4 (7.9)
**Ethnicity - White, n (%)**	1,577 (96.9)	10,614 (97.3)	10,597 (97.1)	10,496 (96.2)	513 (98.1)	7,716 (97.4)	7,673 (96.9)	7,622 (96.2)
**Smoking history, n (%)**								
Current	96 (5.9)	595 (5.5)	533 (4.9)	474 (4.4)	73 (14.1)	614 (7.8)	541 (6.8)	473 (6.0)
Never	990 (61.0)	6,597 (60.6)	6,672 (61.3)	6,880 (63.2)	244 (47.0)	4,058 (51.3)	4,376 (55.4)	4,668 (59.0)
Previous	537 (33.1)	3,694 (33.9)	3,688 (33.9)	3,540 (32.5)	202 (38.9)	3,231 (40.9)	2,985 (37.8)	2,767 (35.0)
**Alcohol consumption, n (%)[1]**								
Never	73 (4.5)	379 (3.5)	292 (2.7)	333 (3.1)	12 (2.3)	123 (1.6)	120 (1.5)	107 (1.4)
Ex-drinker	60 (3.7)	288 (2.7)	246 (2.3)	214 (2.0)	13 (2.5)	198 (2.5)	180 (2.3)	167 (2.1)
Within guidelines	1,129 (69.6)	7,305 (67.4)	7,281 (67.0)	7,269 (66.9)	235 (45.3)	3,305 (41.9)	3,229 (40.9)	3,352 (42.5)
Above guidelines	361 (22.2)	2,874 (26.5)	3,044 (28.0)	3,048 (28.1)	259 (49.9)	4,259 (54.0)	4,360 (55.3)	4,268 (54.1)
**Education, n (%)**								
College	657 (40.4)	4,764 (43.7)	4,920 (45.1)	5,247 (48.1)	218 (41.7)	3,834 (48.4)	3,815 (48.2)	3,962 (50.0)
A/AS level	191 (11.7)	1,541 (14.1)	1,621 (14.9)	1,581 (14.5)	79 (15.1)	917 (11.6)	999 (12.6)	997 (12.6)
O level	381 (23.4)	2,408 (22.1)	2,321 (21.3)	2,264 (20.7)	87 (16.6)	1,329 (16.8)	1,365 (17.2)	1,360 (17.2)
CSE	40 (2.5)	342 (3.1)	400 (3.7)	420 (3.8)	13 (2.5)	227 (2.9)	285 (3.6)	338 (4.3)
NVQ/HND/HNC	64 (3.9)	331 (3.0)	340 (3.1)	289 (2.6)	41 (7.8)	595 (7.5)	566 (7.1)	555 (7.0)
Other	295 (18.1)	1,526 (14.0)	1,310 (12.0)	1,111 (10.2)	85 (16.3)	1,019 (12.9)	890 (11.2)	708 (8.9)
**Fruit and vegetable consumption, n (%)[2]**								
Low	696 (43.4)	4,686 (43.4)	4,721 (43.7)	4,592 (42.5)	254 (49.5)	3,464 (44.4)	3,337 (42.8)	3,254 (41.5)
Moderate	510 (31.8)	3,384 (31.3)	3,301 (30.5)	3,292 (30.5)	121 (23.6)	1,851 (23.7)	1,934 (24.8)	1,866 (23.8)

High	398 (24.8)	2,729 (25.3)	2,793 (25.8)	2,927 (27.1)	138 (26.9)	2,495 (31.9)	2,533 (32.5)	2,723 (34.7)
**Medication, n (%)**								
Cholesterol	222 (13.6)	1,111 (10.2)	716 (6.6)	440 (4.0)	139 (26.6)	1,557 (19.7)	1,088 (13.7)	757 (9.6)
Insulin	8 (0.5)	59 (0.5)	28 (0.3)	42 (0.4)	5 (1.0)	70 (0.9)	47 (0.6)	35 (0.4)
Blood pressure	353 (21.7)	1,601 (14.7)	1,060 (9.7)	696 (6.4)	164 (31.4)	1,704 (21.5)	1,165 (14.7)	767 (9.7)
**Diagnosed cancer, n (%)**	243 (14.9)	1,352 (12.4)	1,148 (10.5)	994 (9.1)	52 (9.9)	603 (7.6)	478 (6.0)	356 (4.5)
**Family history of CVD, n (%)**	999 (61.4)	6,403 (58.7)	5,976 (54.8)	5,665 (51.9)	286 (54.7)	4,258 (53.8)	4,101 (51.8)	3,869 (48.9)
**Light activity (min/day), mean median (SDIQR)**	85 (77)	89.1 (80.4)	88.4 (76.4)	95.9 (75.6)	84.1 (76.4)	89.3 (81.4)	90.9 (75.1)	97.9 (71.2)
**Moderate activity (min/day), median (IQR)mean (SD)**	12.4 (15.7)	21.2 (22)	31.1 (27.2)	41.3 (33.7)	10.6 (15.1)	20 (21)	27.9 (25.1)	37.3 (30.5)
**Sleep duration (min/day), median (IQR)mean (SD)**	437.6 (80.7)	441.3 (74.1)	442.6 (70.8)	441.3 (66.4)	440.6 (79.7)	433.8 (79.6)	434.8 (73.6)	435.2 (70.1)
**glycated haemoglobin (HbA1c), mean (SD)**	35.9 (5.1)	35.3 (4.8)	34.6 (4.2)	34.3 (4.0)	37.4 (8.4)	35.7 (6.3)	34.9 (5.3)	34.4 (4.4)
**HDL (mmol/L), mean (SD)**	1.6 (0.4)	1.6 (0.4)	1.7 (0.4)	1.7 (0.4)	1.3 (0.3)	1.3 (0.3)	1.3 (0.3)	1.4 (0.3)
**LDL (mmol/L), mean (SD)**	3.7 (0.9)	3.7 (0.9)	3.6 (0.8)	3.5 (0.8)	3.5 (0.9)	3.5 (0.8)	3.6 (0.8)	3.6 (0.8)
**Triglycerides (mmol/L), mean (SD)**	1.6 (0.9)	1.5 (0.8)	1.4 (0.8)	1.3 (0.7)	2.1 (1.1)	2.0 (1.1)	1.9 (1.1)	1.7 (1.0)
**Diastolic blood pressure (mmHg), mean (SD)**	82.0 (10.3)	80.8 (10.3)	79.5 (10.4)	78.5 (10.3)	85.1 (10.6)	84.3 (10.2)	83.6 (10.2)	82.5 (10.2)
**Systolic blood pressure (mmHg), mean (SD)**	141.2 (20.5)	137.5 (19.9)	134.5 (19.6)	132.1 (18.9)	146.1 (18.9)	143.4 (18.2)	141.6 (17.7)	139.7 (17.5)
**Body mass index (kg/m2), mean (SD)**	28.1 (5.3)	26.8 (4.7)	25.6 (4.2)	24.5 (3.7)	29.1 (4.4)	27.6 (4.0)	26.8 (3.5)	25.9 (3.2)
**MACE incidence, n (%)**	66 (4.1)	330 (3.0)	198 (1.8)	155 (1.4)	47 (9.0)	511 (6.5)	304 (3.8)	243 (3.1)
**MACE subtypes**								
Myocardial infarction	27 (1.7)	133 (1.0)	69 (0.6)	58 (0.5)	23 (4.4)	243 (3.1)	153 (1.9)	122 (1.5)
Heart Failure	19 (1.2)	100 (0.9)	56 (0.5)	35 (0.3)	14 (2.7)	149 (1.9)	74 (0.9)	48 (0.6)
Stroke[3]	20 (1.2)	97 (0.9)	75 (0.7)	60 (0.5)	10 (1.9)	128 (1.6)	71 (0.9)	70 (0.9)
Haemorrhagic	6 (0.4)	31 (0.3)	16 (0.1)	15 (0.1)	1 (0.2)	31 (0.4)	14 (0.2)	21 (0.3)

Ischemic	13 (0.8)	66 (0.6)	62 (0.6)	44 (0.4)	9 (1.7)	98 (1.2)	57 (0.6)	51 (0.6)

The columns breakdown corresponds to *d*uration of VILPPA bouts. Values represent mean (SD) unless specified otherwise. [1]Alcohol consumption: above guidelines is >14 units per week, where 1 unit = 8 g of ethanol. [2]Fruits and vegetable consumption: low is <5 servings per day, high is >8 servings per day. [3]There were 3 cases in stroke which was undetermined. Thus, they do not add to total stroke incidence.

eTable 2: mean and median bout length of vigorous intermittent lifestyle physical activity (VILPA) in non-exercisers, and vigorous physical activity (VPA) in exercisers). Values represent seconds

Females (n= 13,018)

	Median	Mean	SD
VILPA			
Up to 1 min	20	26.6	15.0
Up to 2 min	30	32.8	23.7
Over 2 min	190	218.7	293.1
VPA			
Up to 1 min	30	38.3	15.6
Up to 2 min	30	47.6	26.9
Over 2 min	220	291.7	376.7

Males (n= 9,350)

	Median	Mean	SD
VILPA			
Up to 1 min	30	38.8	15.7
Up to 2 min	30	47.2	25.7
Over 2 min	190	225.5	319.6
VPA			
Up to 1 min	30	40.1	16.1
Up to 2 min	30	51.7	28.3
Over 2 min	240	362.8	492.5

eTable 3: Sex-specific hazard ratios associated with the minimum dose and median VPA values among non-exercisers for bouts lasting up to 1 minute.

A. All MACE

**Female (n=13,018)**				
**VILPA duration (minutes/day)**	Dose	HR	Lower 95 CI	Upper 95 CI
Minimum dose	1.6	0.70	0.58	0.86
VILPA median	3.4	0.55	0.41	0.75
**Length standardised frequency (1-minute equivalent bouts/day)**				
Minimum dose	2.2	0.50	0.37	0.68
VILPA median	1.4	0.56	0.42	0.75
**Raw Frequency (bouts/day)**				
Minimum dose	9.6	0.63	0.46	0.86
VILPA median	9.3	0.63	0.46	0.87
**Male (n=9,350)**				
**VILPA duration (minutes/day)**				
Minimum dose	2.3	0.89	0.70	1.12
VILPA median	5.6	0.84	0.63	1.12
**Length standardised frequency (1-minute equivalent bouts/day)**				
Minimum dose	1.7	0.85	0.65	1.10
VILPA median	2.2	0.83	0.60	1.10
**Raw Frequency (bouts/day)**				
Minimum dose	4.4	0.86	0.71	1.03
VILPA median	11.4	0.76	0.56	1.02

B. Myocardial infarction

**Female** (n =12,816)				
**VILPA duration (minutes/day)**	Dose	HR	Lower 95 CI	Upper 95 CI
Minimum dose	1.5	0.67	0.50	0.91
VILPA median	3.4	0.49	0.30	0.80
**Length standardised frequency (1-minute equivalent bouts/day)**				
Minimum dose	1.5	0.50	0.31	0.79
VILPA median	1.4	0.51	0.32	0.80
**Raw Frequency (bouts/day)**				
Minimum dose	12.4	0.59	0.36	0.99

VILPA median	9.3	0.67	0.40	1.11
**Male** (n =9,112)				
**VILPA duration (minutes/day)**				
Minimum dose	3.9	0.89	0.59	1.40
VILPA median	5.6	0.87	0.58	1.31
**Length standardised frequency (1-minute equivalent bouts/day)**				
Minimum dose	1.8	0.85	0.59	1.24
VILPA median	2.2	0.84	0.57	1.24
**Raw Frequency (bouts/day)**				
Minimum dose	19.6	0.87	0.57	1.33
VILPA median	11.4	0.92	0.59	1.43

C. Heart failure

**Female** (n =12,783)				
**VILPA duration (minutes/day)**	Dose	HR	Lower 95 CI	Upper 95 CI
Minimum dose	1.2	0.60	0.45	0.81
VILPA median	3.4	0.33	0.18	0.59
**Length standardised frequency (1-minute equivalent bouts/day)**				
Minimum dose	0.6	0.55	0.41	0.74
VILPA median	1.4	0.31	0.18	0.54
**Raw Frequency (bouts/day)**				
Minimum dose	3.1	0.59	0.46	0.76
VILPA median	9.3	0.28	0.16	0.49
**Male** (n = 8,981)				
**VILPA duration (minutes/day)**				
Minimum dose	1.2	0.80	0.61	1.04
VILPA median	5.6	0.61	0.35	1.06
**Length standardised frequency (1-minute equivalent bouts/day)**				
Minimum dose	0.6	0.84	0.65	1.08
VILPA median	2.2	0.68	0.40	1.16
**Raw Frequency (bouts/day)**				
Minimum dose	3.1	0.74	0.58	0.95
VILPA median	11.4	0.49	0.28	0.87

Minimal dose (ED_{50} value): defined as the duration/frequency of VILPA associated with 50% of the optimal risk reduction. The VILPA duration and frequency median values were calculated in the sample excluding participants with zero VILPA. Analyses adjusted for age, light intensity, moderate intensity, VILPA bouts over 1-minute, smoking history, alcohol consumption, accelerometry-derived sleep duration, diet, education, self-reported parental history of CVD, previous incidence of cancer, and self-reported medication use (cholesterol, blood pressure, and diabetes). Raw frequency bouts were additionally adjusted for residual of VILPA bouts of 1 minutes.

eTable 4: Sex-specific hazard ratios associated with the minimum dose and median VPA values among exercisers for bouts lasting up to 1 minute.

A. All MACE

Female (n=34,364)				
**VILPA duration (minutes/day)**	Dose	HR	Lower 95 CI	Upper 95 CI
Minimum dose	2.7	0.84	0.73	0.97
VILPA median	5.1	0.77	0.64	0.94
**Length standardised frequency (1-minute equivalent bouts/day)**				
Minimum dose	1.3	0.82	0.70	0.97
VILPA median	5.1	0.74	0.61	0.90
**Raw Frequency (bouts/day)**				
Minimum dose	6.3	0.80	0.68	0.96
VILPA median	5.1	0.84	0.72	0.96
**Male (n=24,284)**				
**VILPA duration (minutes/day)**				
Minimum dose	3.8	0.79	0.71	0.88
VILPA median	8.1	0.68	0.57	0.80
**Length standardised frequency (1-minute equivalent bouts/day)**				
Minimum dose	1.5	0.78	0.70	0.88
VILPA median	8.1	0.63	0.53	0.75
**Raw Frequency (bouts/day)**				
Minimum dose	6.1	0.74	0.66	0.83
VILPA median	8.1	0.67	0.58	0.79

B. Myocardial infarction

Female (n=33,902)				
**VILPA duration (minutes/day)**	Dose	HR	Lower 95 CI	Upper 95 CI
Minimum dose	3.5	0.69	0.53	0.89
VILPA median	5.1	0.62	0.45	0.84
**Length standardised frequency (1-minute equivalent bouts/day)**				
Minimum dose	1.4	0.69	0.53	0.89
VILPA median	2.1	0.61	0.44	0.85
**Raw Frequency (bouts/day)**				
Minimum dose	5.8	0.65	0.51	0.82

VILPA median	11.9	0.45	0.31	0.66
**Male (n=23,720)**				
**VILPA duration (minutes/day)**				
Minimum dose	2.2	0.83	0.73	0.95
VILPA median	8.3	0.66	0.50	0.87
**Length standardised frequency (1-minute equivalent bouts/day)**				
Minimum dose	0.8	0.83	0.74	0.95
VILPA median	3.2	0.66	0.50	0.87
**Raw Frequency (bouts/day)**				
Minimum dose	4.1	0.74	0.65	0.85
VILPA median	15	0.49	0.35	0.68

C. Heart failure

**Female (n=33,825)**				
**VILPA duration (minutes/day)**	Dose	HR	Lower 95 CI	Upper 95 CI
Minimum dose	4.5	0.71	0.49	1.04
VILPA median	5.1	0.70	0.47	1.03
**Length standardised frequency (1-minute equivalent bouts/day)**				
Minimum dose	1.8	0.67	0.47	0.96
VILPA median	2.1	0.65	0.44	0.95
**Raw Frequency (bouts/day)**				
Minimum dose	10.6	0.73	0.47	1.13
VILPA median	5.1	0.84	0.65	1.10
**Male (n=23,464)**				
**VILPA duration (minutes/day)**				
Minimum dose	5.5	0.65	0.47	0.89
VILPA median	8.3	0.58	0.40	0.83
**Length standardised frequency (1-minute equivalent bouts/day)**				
Minimum dose	2.5	0.65	0.45	0.94
VILPA median	3.2	0.62	0.43	0.90
**Raw Frequency (bouts/day)**				
Minimum dose	8.6	0.65	0.47	0.90
VILPA median	8.3	0.66	0.48	0.91

Minimal dose (ED_{50} value): defined as the duration/frequency of VILPA associated with 50% of the optimal risk reduction. The VILPA duration and frequency median values were calculated in the sample excluding participants with zero VILPA. Analyses adjusted for age, light intensity, moderate intensity, VILPA bouts over 1-minute,

smoking history, alcohol consumption, accelerometry-derived sleep duration, diet, education, self-reported parental history of CVD, previous incidence of cancer, and self-reported medication use (cholesterol, blood pressure, and diabetes). Raw frequency bouts were additionally adjusted for residual of VILPA bouts of 1 minutes.

eTable 5: Sex-specific e-values for minimum dose and median VILPA values for MACE and its subtypes among non-exercisers for bouts lasting up to 1 minute.

D. All MACE

Female (n=13,018)	
**VILPA duration (minutes/day)**	E-value
Minimum dose	2.21 (1.60)
VILPA median	3.04 (2.00)
**Length standardised frequency (1-minute equivalent bouts/day)**	
Minimum dose	3.41 (2.30)
VILPA median	2.97 (2.00)
**Raw Frequency (bouts/day)**	
Minimum dose	2.55 (1.60)
VILPA median	2.55 (1.56)
**Male (n=9,350)**	
**VILPA duration (minutes/day)**	
Minimum dose	1.49 (1.00)
VILPA median	1.67 (1.00)
**Length standardised frequency (1-minute equivalent bouts/day)**	
Minimum dose	1.63 (1.00)
VILPA median	1.70 (1.00)
**Raw Frequency (bouts/day)**	
Minimum dose	1.60 (1.00)
VILPA median	1.96 (1.00)

E. Myocardial infarction

Female (n=12,816)	
**VILPA duration (minutes/day)**	E-value
Minimum dose	2.34 (1.42)
VILPA median	3.49 (1.80)
**Length standardised frequency (1-minute equivalent bouts/day)**	
Minimum dose	3.41 (1.85)
VILPA median	3.33 (1.81)
**Raw Frequency (bouts/day)**	
Minimum dose	2.78 (0.99)
VILPA median	2.34 (1.00)

**Male (n=9,112)**	
**VILPA duration (minutes/day)**	
Minimum dose	1.50 (1.00)
VILPA median	1.56 (1.00)
**Length standardised frequency (1-minute equivalent bouts/day)**	
Minimum dose	1.63 (1.00)
VILPA median	1.67 (1.00)
**Raw Frequency (bouts/day)**	
Minimum dose	1.56 (1.00)
VILPA median	1.39 (1.00)

F. Heart failure

**Female (n=12,783)**	
**VILPA duration (minutes/day)**	E-value
Minimum dose	2.72 (1.77)
VILPA median	5.51 (2.78)
**Length standardised frequency (1-minute equivalent bouts/day)**	
Minimum dose	3.04 (2.04)
VILPA median	5.91 (3.12)
**Raw Frequency (bouts/day)**	
Minimum dose	2.78 (1.96)
VILPA median	6.60 (3.50)
**Male (n=8,981)**	
**VILPA duration (minutes/day)**	
Minimum dose	1.81 (1.00)
VILPA median	2.66 (1.00)
**Length standardised frequency (1-minute equivalent bouts/day)**	
Minimum dose	1.67 (1.00)
VILPA median	2.30 (1.00)
**Raw Frequency (bouts/day)**	
Minimum dose	2.04 (1.29)
VILPA median	3.49 (1.56)

eTable 6: Sex-specific e-values for minimum dose and median VPA values for MACE and its subtypes among exercisers for bouts lasting up to 1 minute.

A. All MACE

Female (n=34,364)	
**VILPA duration (minutes/day)**	E-value
Minimum dose	1.67 (1.20)
VILPA median	1.92 (1.32)
**Length standardised frequency (1-minute equivalent bouts/day)**	
Minimum dose	1.73 (1.21)
VILPA median	2.04 (1.46)
**Raw Frequency (bouts/day)**	
Minimum dose	1.81 (1.25)
VILPA median	1.67 (1.25)
**Male (n=24,284)**	
**VILPA duration (minutes/day)**	
Minimum dose	1.85 (1.53)
VILPA median	2.30 (1.81)
**Length standardised frequency (1-minute equivalent bouts/day)**	
Minimum dose	1.88 (1.53)
VILPA median	2.34 (1.78)
**Raw Frequency (bouts/day)**	
Minimum dose	2.04 (1.70)
VILPA median	2.35 (1.85)

B. Myocardial infarction

Female (n=33,902)	
**VILPA duration (minutes/day)**	E-value
Minimum dose	2.60 (1.51)
VILPA median	2.61 (1.67)
**Length standardised frequency (1-minute equivalent bouts/day)**	
Minimum dose	2.26 (1.50)
VILPA median	2.66 (1.63)
**Raw Frequency (bouts/day)**	
Minimum dose	2.44 (1.74)
VILPA median	3.87 (2.40)

**Male (n=23,720)**	
**VILPA duration (minutes/day)**	
Minimum dose	1.70 (1.29)
VILPA median	2.40 (1.56)
**Length standardised frequency (1-minute equivalent bouts/day)**	
Minimum dose	1.70 (1.29)
VILPA median	2.40 (1.56)
**Raw Frequency (bouts/day)**	
Minimum dose	2.04 (1.63)
VILPA median	3.49 (2.30)

C. Heart failure

**Female (n=33,825)**	
**VILPA duration (minutes/day)**	E-value
Minimum dose	2.17 (1.00)
VILPA median	2.21 (1.00)
**Length standardised frequency (1-minute equivalent bouts/day)**	
Minimum dose	2.35 (1.25)
VILPA median	2.44 (1.29)
**Raw Frequency (bouts/day)**	
Minimum dose	2.08 (1.00)
VILPA median	1.67 (1.00)
**Male (n=23,464)**	
**VILPA duration (minutes/day)**	
Minimum dose	2.45 (1.50)
VILPA median	2.84 (1.70)
**Length standardised frequency (1-minute equivalent bouts/day)**	
Minimum dose	2.45 (1.32)
VILPA median	2.61 (1.46)
**Raw Frequency (bouts/day)**	
Minimum dose	2.45 (1.46)
VILPA median	2.40 (1.43)

eTable 7: Covariate definitions

Variable	Definition	UK Biobank field ID (if applicable)
Age	Continuous	34, 52, accelerometer date-timestamp
Sex	Female/Male	31
Ethnicity	White/Others ()	
Light intensity physical activity	Standing utilitarian movements, slow walking (<3 METs)	Derived from accelerometer data (see Online Methods)
Moderate intensity physical activity	Brisk walking, energetic activities (≥3 to <6 METs)	Derived from accelerometer data (see Online Methods)
Frequency/duration of bouts above the specified bout length (> 1 or > 2 minutes) in each frequency/duration analysis	Number of bouts above the specified bout in the analysis. Eg, for the analysis of bouts lasting up to 2 minutes in duration, this variable contained bouts/duration that were more than 2 minutes in duration	Derived from accelerometer data (see Methods)
Smoking status	Never, past, current	20116
Alcohol consumption	Never, ex-drinker, within guidelines, above guidelines	20117, 1558
Sleep duration	Hours spent sleeping	Derived from accelerometer data (see Methods)
Diet	Fruits and vegetables servings/day, categorised as low (<5 servings/day), moderate (5 to 8 servings/day) and high (>8 servings/day)	1309, 1319, 1289, 1299
Prevalent cancer	Identified by self-report and cancer registry	20001, 100092
Education	College/University; A/AS level; O levels; CSE; NVQ/HND/HNC; other	6138
Parental history of CVD	Self-reported mother or father diagnosed with heart disease or stroke	20107, 20110
Use of medication (cholesterol, blood pressure and diabetes)	Yes/No	6177, 6153

eTable 8: Multiplicative interaction test between age and daily VILPA duration bouts lasting up to 1 minute (minutes/day) for MACE and its subtypes.

Outcome	Age*VILPA, p-values	
	Female	Male
MACE	0.437	0.941
Myocardial infarction	0.811	0.713
Heart failure	0.172	0.480
Stroke	0.962	0.771

The models were adjusted for sex (in the total sample only)*, light intensity, moderate intensity, VILPA bouts over 1-minute, smoking history, alcohol consumption, accelerometry-derived sleep duration, diet, education, ethnicity, self-reported parental history of CVD, previous incidence of cancer, and self-reported medication use (cholesterol, blood pressure, and diabetes).

eTable 9: Interaction test between sex and daily VILPA duration bouts lasting up to 1 minute (minutes/day) for MACE and its subtypes.

	Sex*VILPA		
Outcome	Multiplicative interaction		Additive relative excess risk due to interaction (RERI)
	HR (95% CI)	p-values	HR (95% CI)
MACE	1.05 (1.01, 1.08)	0.006	-0.47 (-0.69, -0.25)
Myocardial infarction	1.06 (1.01, 1.11)	0.031	0.15 (-0.29, 0.59)
Heart failure	1.11 (1.03, 1.96)	0.008	-0.95 (-1.29, -0.61)
Stroke	0.99 (0.94, 1.05)	0.742	-0.50 (-0.91, -0.082)

The models were adjusted for age, light intensity, moderate intensity, VILPA bouts over 1-minute, smoking history, alcohol consumption, accelerometry-derived sleep duration, diet, education, ethnicity, self-reported parental history of CVD, previous incidence of cancer, and self-reported medication use (cholesterol, blood pressure, and diabetes).

eTable 10: STROBE Statement

	Item No	Recommendation	Page No
**Title and abstract**	1	(*a*) Indicate the study's design with a commonly used term in the title or the abstract	1
		(*b*) Provide in the abstract an informative and balanced summary of what was done and what was found	3
**Introduction**			
Background/rationale	2	Explain the scientific background and rationale for the investigation being reported	5-6
Objectives	3	State specific objectives, including any prespecified hypotheses	6
**Methods**			
Study design	4	Present key elements of study design early in the paper	6-7
Setting	5	Describe the setting, locations, and relevant dates, including periods of recruitment, exposure, follow-up, and data collection	6-7
Participants	6	(*a*) *Cohort study*—Give the eligibility criteria, and the sources and methods of selection of participants. Describe methods of follow-up<br>*Case-control study*—Give the eligibility criteria, and the sources and methods of case ascertainment and control selection. Give the rationale for the choice of cases and controls<br>*Cross-sectional study*—Give the eligibility criteria, and the sources and methods of selection of participants	6-7
		(*b*) *Cohort study*—For matched studies, give matching criteria and number of exposed and unexposed<br>*Case-control study*—For matched studies, give matching criteria and the number of controls per case	
Variables	7	Clearly define all outcomes, exposures, predictors, potential confounders, and effect modifiers. Give diagnostic criteria, if applicable	7-8
Data sources/ measurement	8*	For each variable of interest, give sources of data and details of methods of assessment (measurement). Describe comparability of assessment methods if there is more than one group	8
Bias	9	Describe any efforts to address potential sources of bias	8
Study size	10	Explain how the study size was arrived at	10-11
Quantitative variables	11	Explain how quantitative variables were handled in the analyses. If applicable, describe which groupings were chosen and why	7-8
Statistical methods	12	(*a*) Describe all statistical methods, including those used to control for confounding	8-10
		(*b*) Describe any methods used to examine subgroups and interactions	9-10
		(*c*) Explain how missing data were addressed	6
		(*d*) *Cohort study*—If applicable, explain how loss to follow-up was addressed<br>*Case-control study*—If applicable, explain how matching of cases and controls was addressed<br>*Cross-sectional study*—If applicable, describe analytical methods taking account of sampling strategy	6-7
		(*e*) Describe any sensitivity analyses	10
**Results**			
Participants	13*	(a) Report numbers of individuals at each stage of study—eg numbers potentially eligible, examined for eligibility, confirmed	10-11

		eligible, included in the study, completing follow-up, and analysed	
		(b) Give reasons for non-participation at each stage	10-11
		(c) Consider use of a flow diagram	eFigure 1 and 2
Descriptive data	14*	(a) Give characteristics of study participants (eg demographic, clinical, social) and information on exposures and potential confounders	10-11
		(b) Indicate number of participants with missing data for each variable of interest	eFigure 1 and 2
		(c) *Cohort study*—Summarise follow-up time (eg, average and total amount)	11
Outcome data	15*	*Cohort study*—Report numbers of outcome events or summary measures over time	11
		Case-control study—Report numbers in each exposure category, or summary measures of exposure	
		Cross-sectional study—Report numbers of outcome events or summary measures	
Main results	16	(*a*) Give unadjusted estimates and, if applicable, confounder-adjusted estimates and their precision (eg, 95% confidence interval). Make clear which confounders were adjusted for and why they were included	10-13
		(*b*) Report category boundaries when continuous variables were categorized	11-13
		(*c*) If relevant, consider translating estimates of relative risk into absolute risk for a meaningful time period	11-14
Other analyses	17	Report other analyses done—eg analyses of subgroups and interactions, and sensitivity analyses	14
**Discussion**			
Key results	18	Summarise key results with reference to study objectives	15-16
Limitations	19	Discuss limitations of the study, taking into account sources of potential bias or imprecision. Discuss both direction and magnitude of any potential bias	17
Interpretation	20	Give a cautious overall interpretation of results considering objectives, limitations, multiplicity of analyses, results from similar studies, and other relevant evidence	17
Generalisability	21	Discuss the generalisability (external validity) of the study results	17-18
**Other information**			
Funding	22	Give the source of funding and the role of the funders for the present study and, if applicable, for the original study on which the present article is based	18

eTable 11: Estimated maximal oxygen consumption, and average oxygen consumption and relative intensity of VILPA bouts (up to 1 minute) among 1,588 males non-exercisers UK Biobank participants with valid accelerometry and ergometer data (see **eText 3).**

**VO_2 max**	
Females	25.56 (6.82) ml/kg/min<br>7.30 (1.95) METs
Males	32.32 (8.78) ml/kg/min<br>9.24 (2.51) METs
**Average VO_2 during VILPA bouts**	
Females	21.05 (3.56) ml/kg/min<br>6.04 (1.02) METs
Males	21.74 (5.32) ml/kg/min<br>6.21 (1.52) METs
**Average relative intensity (%VO2max) during VILPA bouts**	
Females	83.2 (18.2) %
Males	70.5 (22.1) %

eFigure 1: Flow diagram of non-exerciser participants

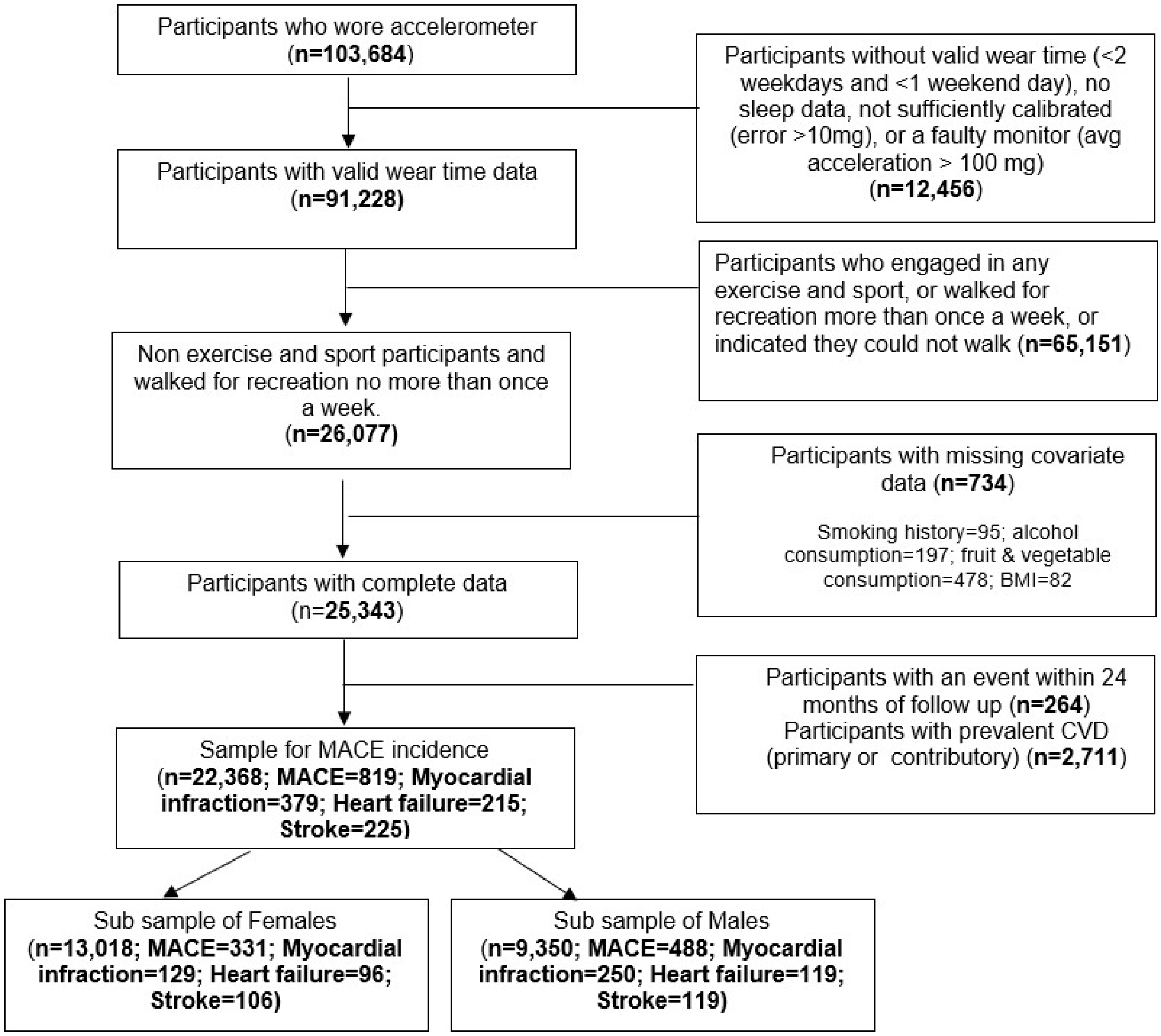

eFigure 2: Flow diagram of exercisers participants

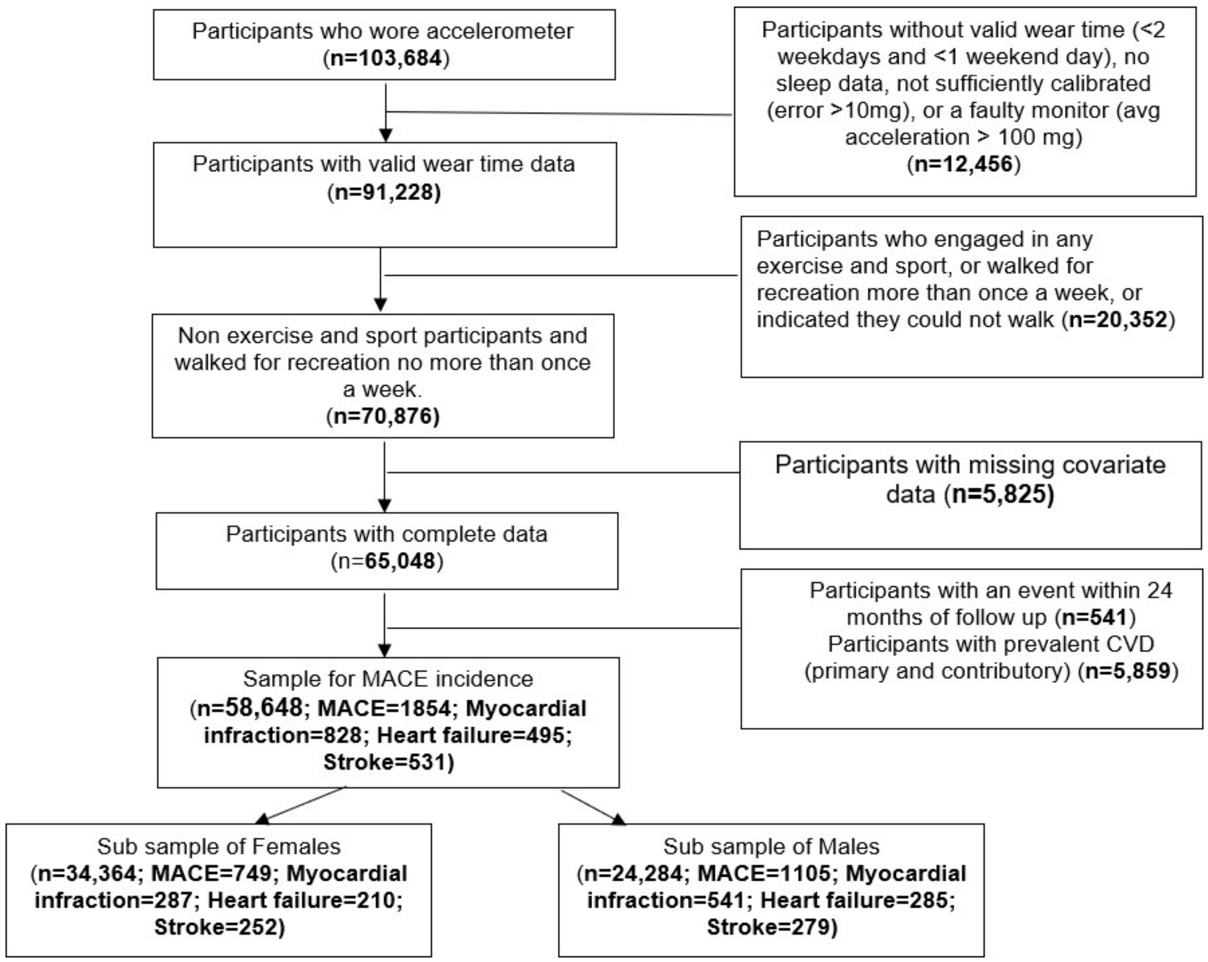

eFigure 3: Sex-specific adjusted absolute risk estimates of daily VILPA duration for MACE and its subtypes, bouts lasting up to 1 minute (minutes/day).

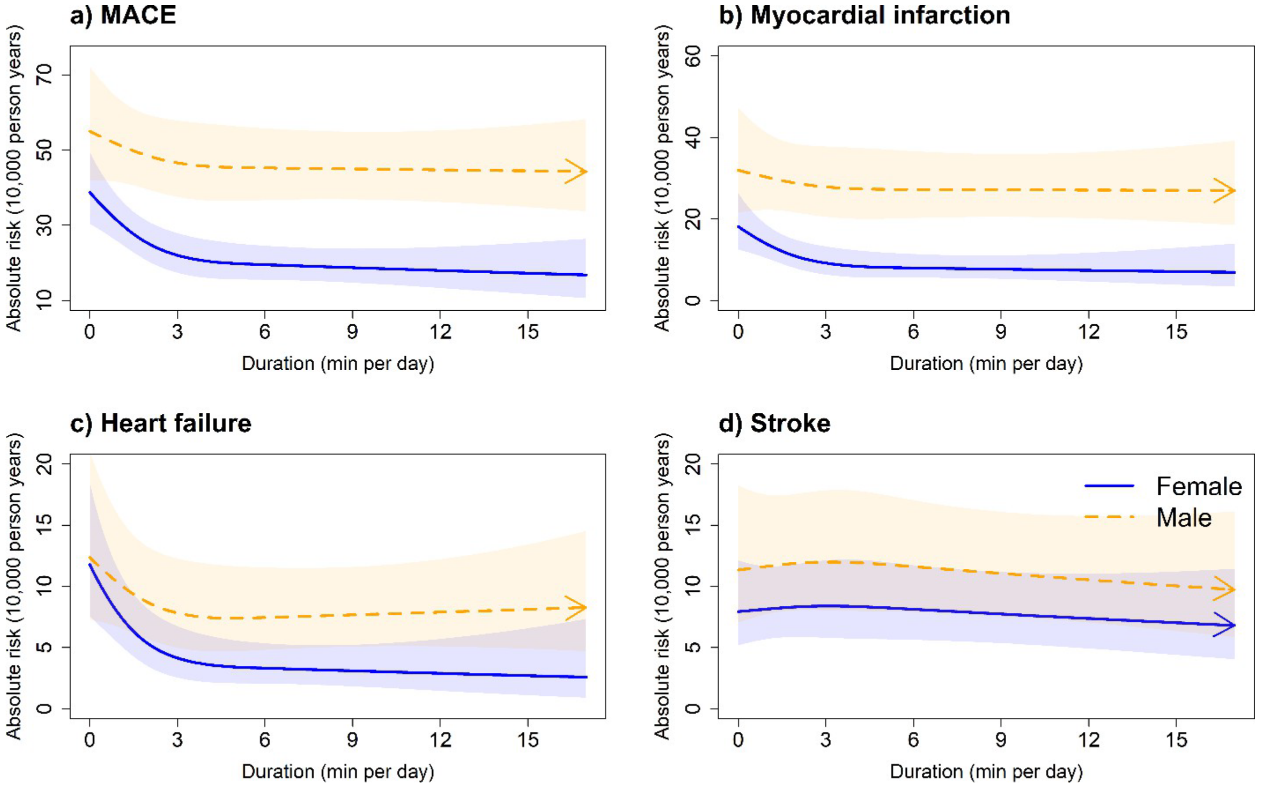

Legend: Adjusted for age, light intensity, moderate intensity, VILPA bouts over 1-minute, smoking history, alcohol consumption, accelerometer estimated sleep duration, diet, education, ethnicity, self-reported parental history of CVD, previous incidence of cancer, and self-reported medication use (cholesterol, blood pressure, and diabetes). **Panel A:** MACE: n = 22,368; events: all MACE = 819 (female/male = 331/488), **Panel B:** myocardial infarction: n = 21,928; events = 379 (female/male = 129/250). **Panel C:** heart failure: n = 21,764; events = 215 (female/male = 96/119). **Panel D:** stroke: n = 21,774; events = 225 (female/male = 106/119).

eFigure 4: Adjusted sex-specific dose response curves of VILPA frequency for MACE and its sub- types, raw VILPA bouts lasting up to 1 minutes (bouts/day).

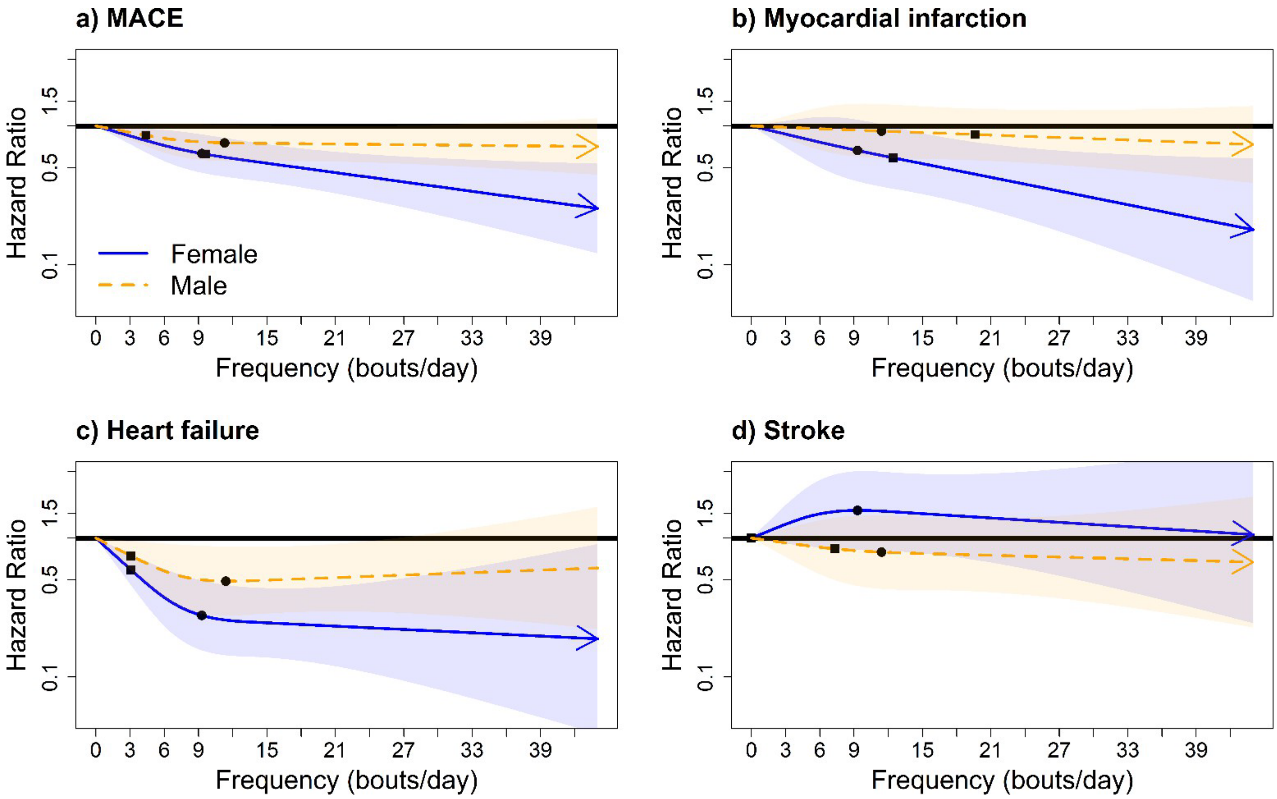

Legend: Adjusted for age, light intensity, moderate intensity, VILPA bouts over 1-minute, smoking history, alcohol consumption, accelerometry-derived sleep duration, diet, education, ethnicity, self-reported parental history of CVD, previous incidence of cancer, self-reported medication use (cholesterol, blood pressure, and diabetes) and residual of VILPA duration of 1-minute bouts. **Panel A:** all MACE: n = 22,368; events: 819 (f emale/male = 331/488), **Panel B:** myocardial infarction: n = 21,928; events = 379 (female/male = 129/250). **Panel C:** heart failure: n = 21,764; events = 215 (female/male = 96/119). **Panel D:** stroke: n = 21,774; events = 225 (female/male = 106/119). Diamond, minimal dose, as indicated by the ED_{50} statistic which estimates the daily duration of VILPA associated with 50% of optimal risk reduction. Circle, HR associated with the median VILPA value (see eTable 3 for the list of values).

eFigure 5: Adjusted sex-specific dose response curves of daily VILPA duration for MACE and its sub-types, bouts lasting up to 2 minutes (minutes/day).

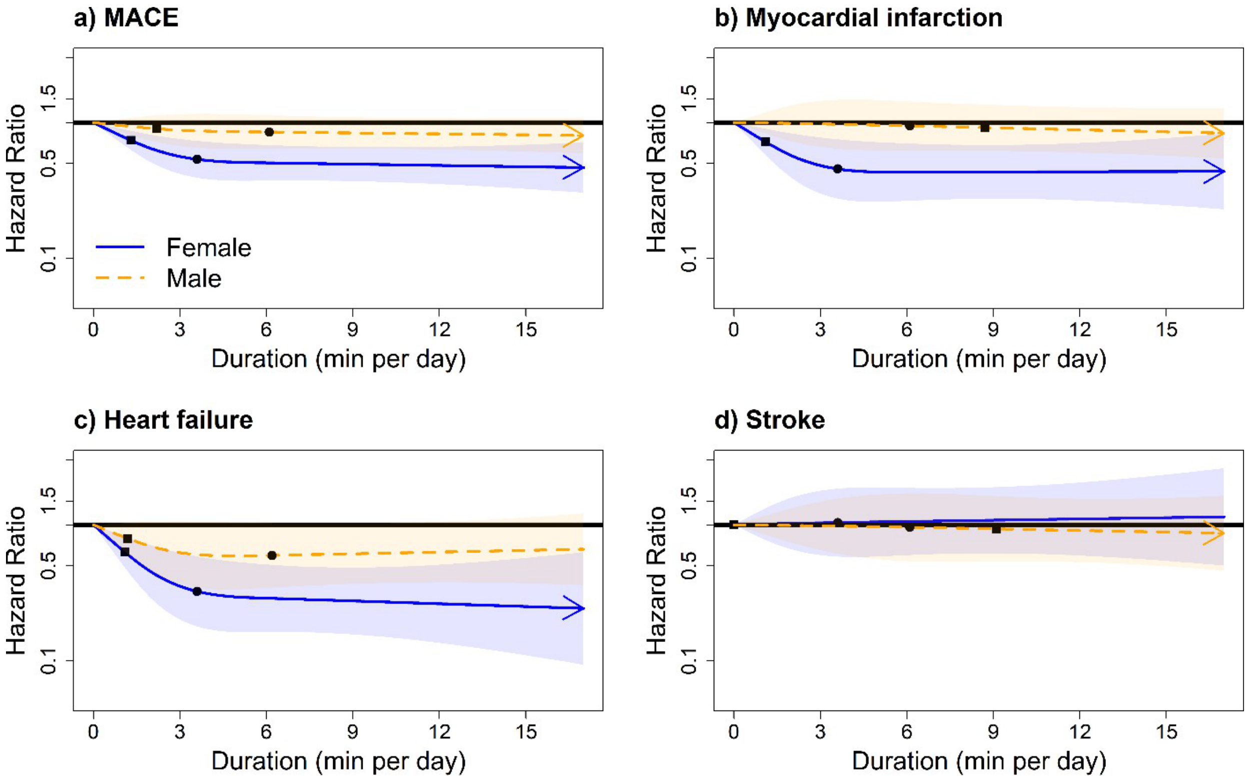

Legend: Adjusted for age, light intensity, moderate intensity, VILPA bouts over 1-minute, smoking history, alcohol consumption, accelerometry-derived sleep duration, diet, education, ethnicity, self-reported parental history of CVD, previous incidence of cancer, and self-reported medication use (cholesterol, blood pressure, and diabetes). **Panel A:** all MACE: n = 22,368; events: 819 (female/male = 331/488), **Panel B:** myocardial infarction: n = 21,928; events = 379 (female/male = 129/250). **Panel C:** heart failure: n = 21,764; events = 21 5 (female/male = 96/119). **Panel D:** stroke: n = 21,774; events = 225 (female/male = 106/119). Diamond, minimal dose, as indicated by the ED_{50} statistic which estimates the daily duration of VILPA associated with 50 % of optimal risk reduction. Circle, HR associated with the median VILPA value.

eFigure 6: Sex-specific adjusted dose response curves of frequency of vigorous physical activity (VPA) in exercisers for MACE and its subtypes for length-standardised bouts lasting 1 minute.

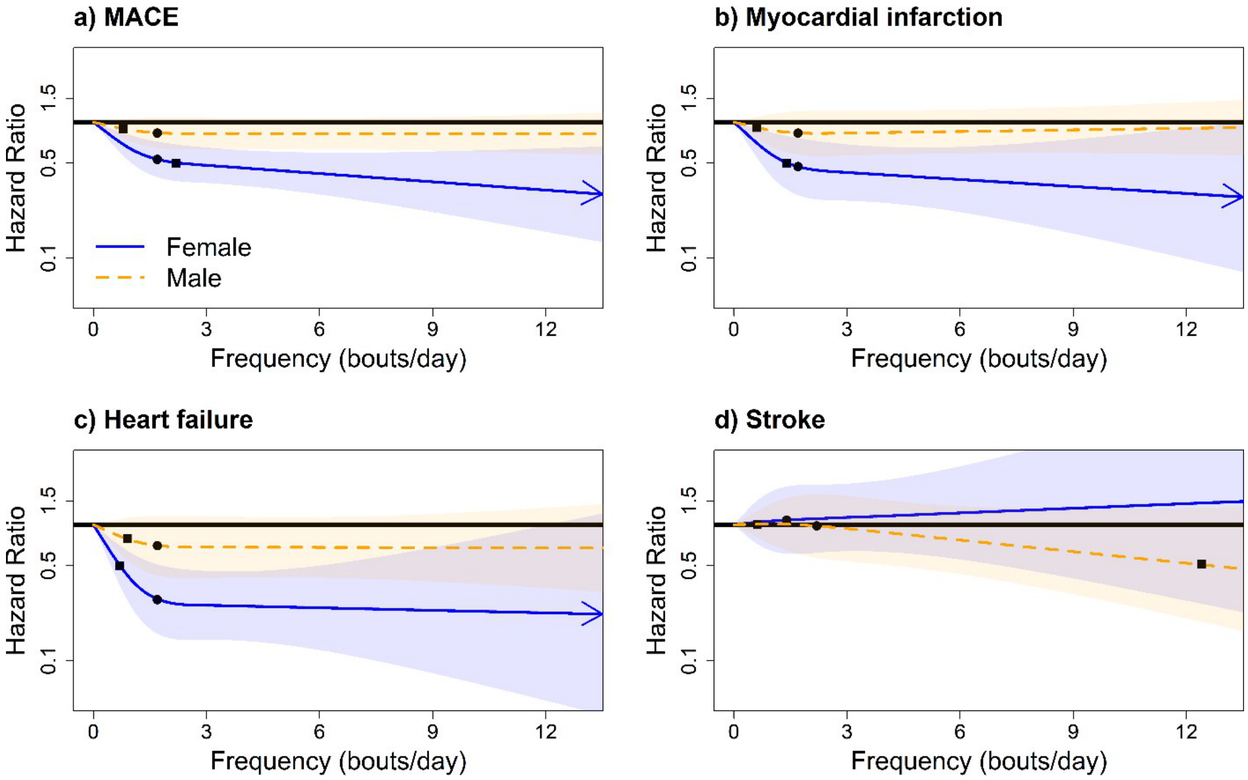

Legend: Adjusted for sex, age, light intensity, moderate intensity, VILPA bouts over 1-minute, smoking hist ory, alcohol consumption, accelerometry-derived sleep duration, diet, education, ethnicity, self-reported pare ntal history of CVD, previous incidence of cancer, and self-reported medication use (cholesterol, blood press ure, and diabetes). The range was capped at the 97.5 percentile to minimize the influence of sparse data. **Pan el A:** MACE: n = 58,648; events: 1854 (female/male = 749/1105), **Panel B:** myocardial infarction: n = 57,62 2; events = 828 (female/male = 287/541). **Panel C:** heart failure: n = 57,289; events = 495 (female/male = 21 0/285). **Panel D:** stroke: n = 57,325; events = 531 (female/male = 252/279). Diamond, minimal dose, as indicated by the ED_{50} statistic which estimates the daily duration of VILPA associated with 50% of optimal risk reduction. Circle, HR associated with the median VILPA value (see eTable 3 for the list of values).

eFigure 7: Sex-specific adjusted dose response curves of frequency of vigorous physical activity (VPA) in exercisers for MACE and its subtypes for raw bouts lasting up to 1 minute .

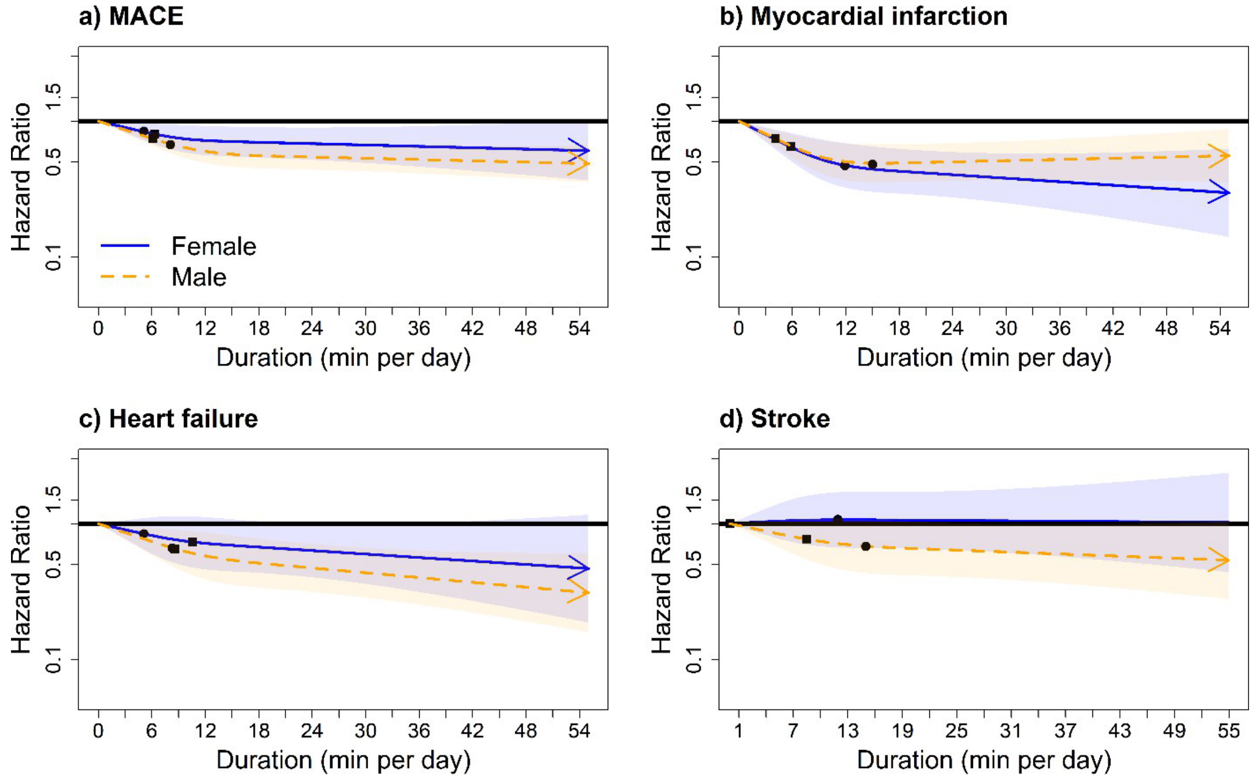

Legend: Adjusted for sex, age, light intensity, moderate intensity, VILPA bouts over 1-minute, smoking hist ory, alcohol consumption, accelerometry-derived sleep duration, diet, education, ethnicity, self-reported pare ntal history of CVD, previous incidence of cancer, self-reported medication use (cholesterol, blood pressure, and diabetes) and residual of VILPA duration of 1-minute bouts. **Panel A:** MACE: n = 58,648; events: 1854 (female/male = 749/1105), **Panel B:** myocardial infarction: n = 57,622; events = 828 (female/male = 287/54 1). **Panel C:** heart failure: n = 57,289; events = 495 (female/male = 210/285). **Panel D:** stroke: n = 57,325; e vents = 531 (female/male = 252/279). Diamond, minimal dose, as indicated by the ED_{50} statistic which estim ates the daily duration of VILPA associated with 50% of optimal risk reduction. Circle, HR associated with t he median VILPA value (see eTable 4 for the list of values).

eFigure 8: Adjusted Sex-specific dose response curves of daily VILPA duration for MACE and its subtypes, with additional adjustment for glycated haemoglobin (HbA1c), low-density lipoprotein (LDL), high-density lipoprotein (HDL), triglycerides, systolic blood pressure, diastolic blood pressure, and body mass index; bouts lasting up to 1 minute (minutes/day).

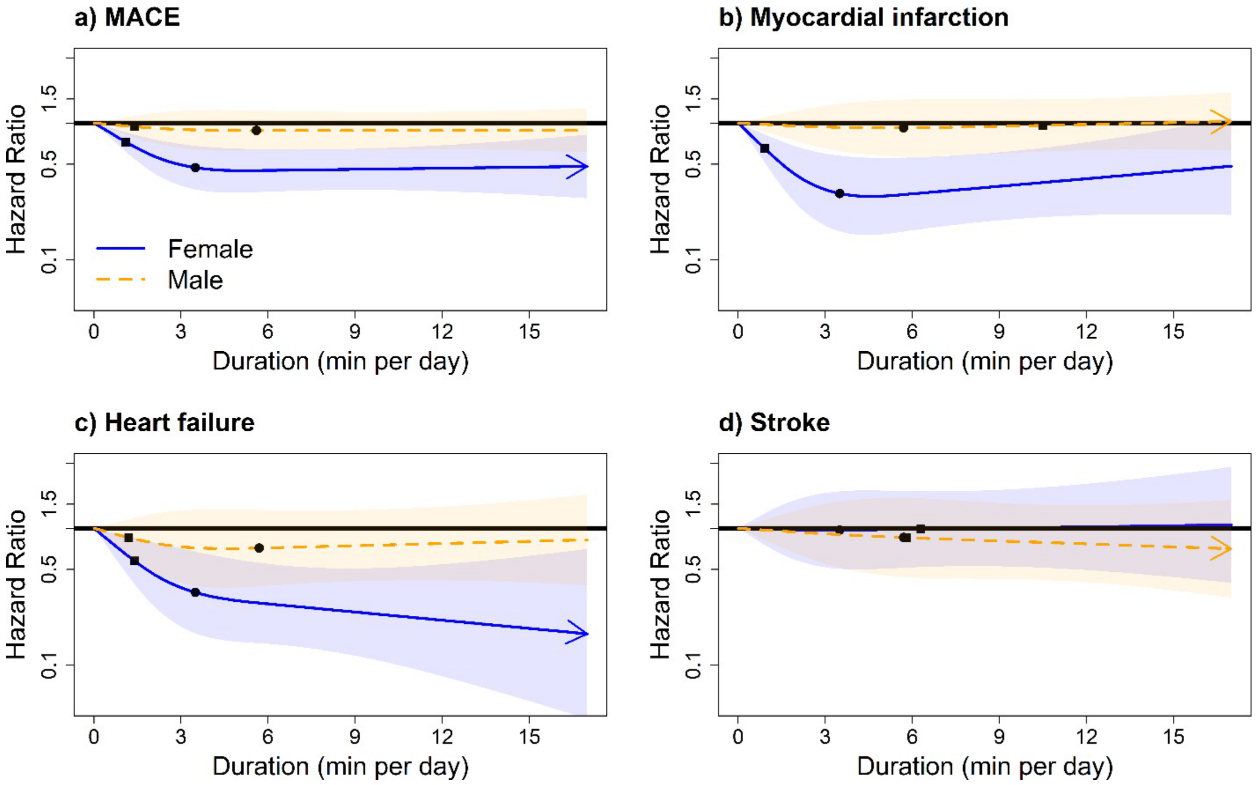

Legend: Adjusted for age, light intensity, moderate intensity, VILPA bouts over 1-minute, smoking history, alcohol consumption, accelerometry-derived sleep duration, diet, education, self-reported parental history of CVD, previous incidence of cancer, self-reported medication use (cholesterol, blood pressure, and diabetes), glycated haemoglobin (HbA1c), low-density lipoprotein (LDL), high-density lipoprotein (HDL), triglyceride s, systolic blood pressure, diastolic blood pressure, and body mass index. **Panel A:** all MACE: n = 22,289; e vents: 817 (female/male = 329/488), **Panel B:** myocardial infarction: n = 21,850; events = 378 (female/male = 128/250). **Panel C:** heart failure: n = 21,687; events = 215 (female/male = 96/119). **Panel D:** stroke: n = 2 0,895; events = 224 (female/male = 105/119). Diamond, minimal dose, as indicated by the ED_{50} statistic which estimates the daily duration of VILPA associated with 50% of optimal risk reduction. Circle, HR associated with the median VILPA value.

eFigure 9: Adjusted Sex-specific dose response curves of daily VILPA duration for MACE and its subtypes, after excluding participants with poor health or a BMI below 18.5 kg/m^2 or current smokers.

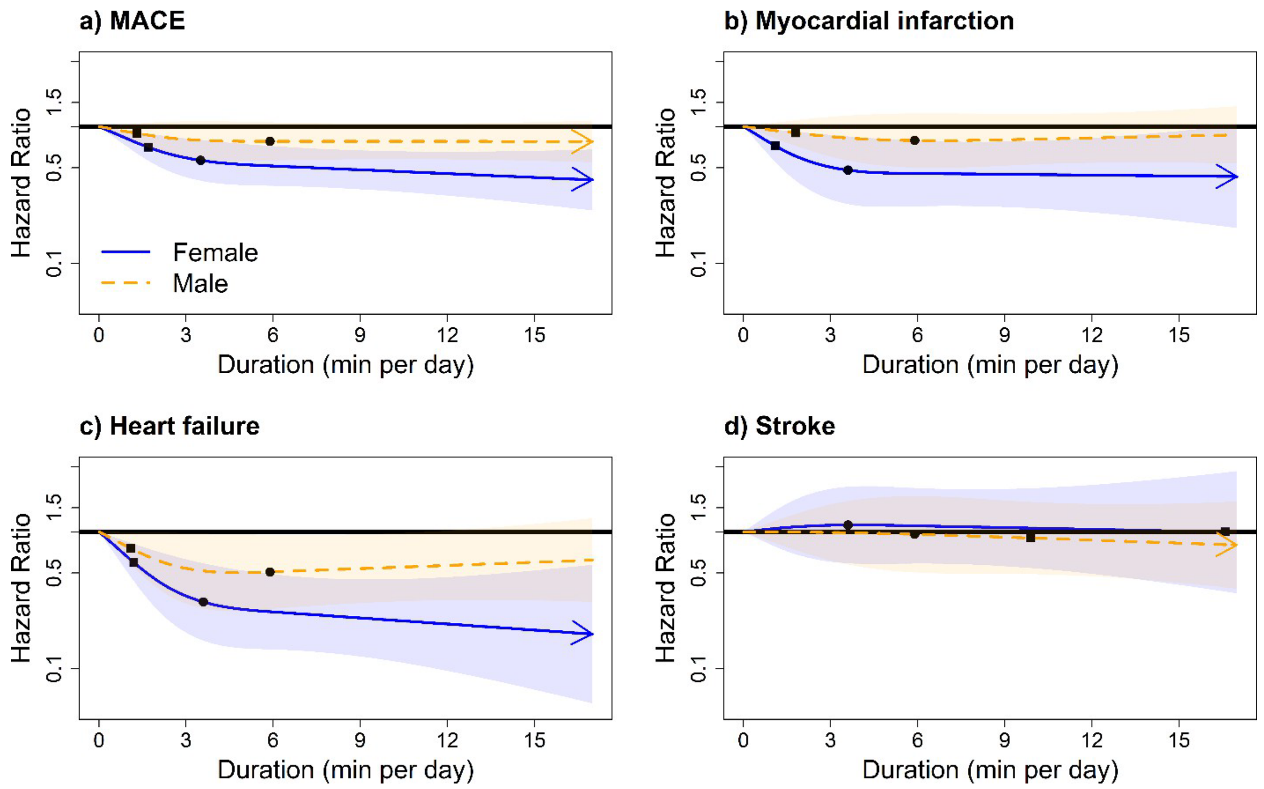

Legend: Adjusted for age, light intensity, moderate intensity, VILPA bouts over 1-minute, smoking history, alcohol consumption, accelerometry-derived sleep duration, diet, education, self-reported parental history of CVD, previous incidence of cancer, and self-reported medication use (cholesterol, blood pressure, and diabet es). **Panel A:** all MACE: n = 19,471; events: 661 (female/male = 271/390), **Panel B:** myocardial infarction: n = 19,116; events = 306 (female/male = 105/201). **Panel C:** heart failure: n = 18,983; events = 173 (female/ male = 80/93). **Panel D:** stroke: n = 18,348; events = 182 (female/male = 86/96). Diamond, minimal dose, as indicated by the ED_{50} statistic which estimates the daily duration of VILPA associated with 50% of optimal risk reduction. Circle, HR associated with the median VILPA value.

eFigure 10: Adjusted sex-specific dose response curves of VILPA duration for MACE and its sub-types, VILPA bouts lasting up to 1 minutes (bouts/day), with reference as 15th percentile for VILPA duration (0.625 mins/day).

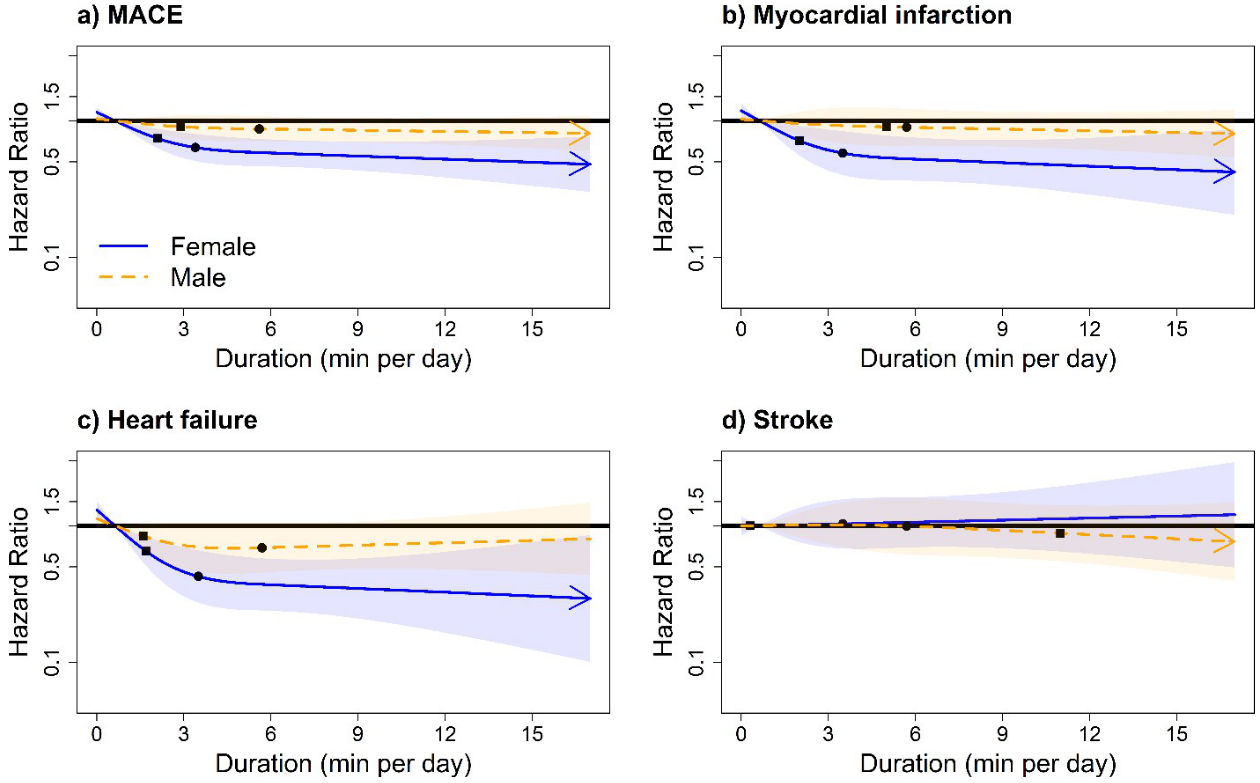

Legend: Adjusted for age, light intensity, moderate intensity, VILPA bouts over 1-minute, smoking history, alcohol consumption, accelerometry-derived sleep duration, diet, education, self-reported parental history of CVD, previous incidence of cancer, and self-reported medication use (cholesterol, blood pressure, and diabet es). **Panel A:** all MACE: n = 19,471; events: 661 (female/male = 271/390), **Panel B:** myocardial infarction: n = 19,116; events = 306 (female/male = 105/201). **Panel C:** heart failure: n = 18,983; events = 173 (female/ male = 80/93). **Panel D:** stroke: n = 18,348; events = 182 (female/male = 86/96). Diamond, minimal dose, as indicated by the ED_{50} statistic which estimates the daily duration of VILPA associated with 50% of optimal r isk reduction. Circle, HR associated with the median VILPA value.

eFigure 11: Adjusted sex-specific dose response curves of VILPA duration for MACE and its sub-types, VILPA bouts lasting up to 1 minutes (bouts/day), excluding frail participants (n = 1,924).

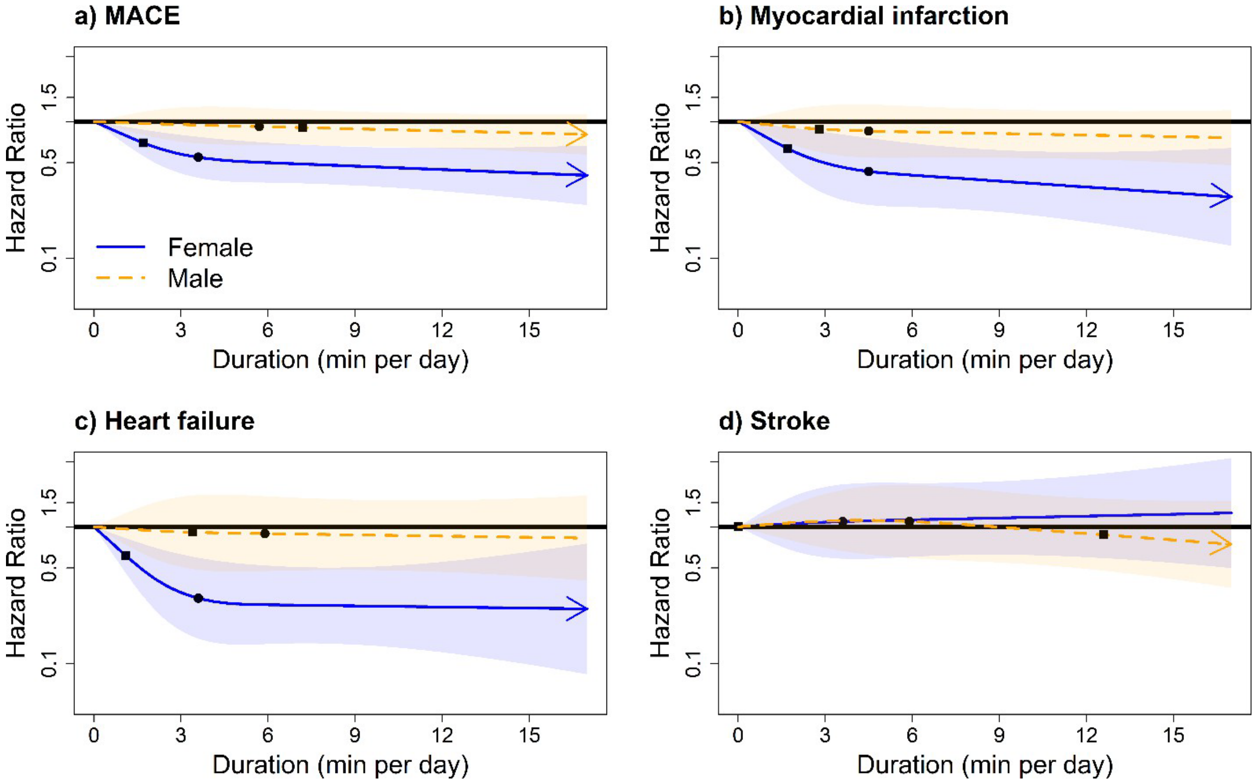

Legend: Adjusted for age, light intensity, moderate intensity, VILPA bouts over 1-minute, smoking history, alcohol consumption, accelerometry-derived sleep duration, diet, education, self-reported parental history of CVD, previous incidence of cancer, and self-reported medication use (cholesterol, blood pressure, and diabet es). **Panel A:** all MACE: n = 19,465 events: 697 (female/male = 271/426), **Panel B:** myocardial infarction: n = 19,096; events = 328 (female/male = 105/223). **Panel C:** heart failure: n = 18,940; events = 172 (female/m ale = 73/99). **Panel D:** stroke: n = 18,965; events = 197 (female/male = 93/104). Diamond, minimal dose, as indicated by the ED_{50} statistic which estimates the daily duration of VILPA associated with 50% of optimal risk reduction. Circle, HR associated with the median VILPA value.

eFigure 12: Age-stratified and sex-specific dose response curves of daily VILPA duration for MACE and its subtypes for bouts lasting up to 1 minute (minutes/day).

A) Age up to median (42.9 - 62.4 years)

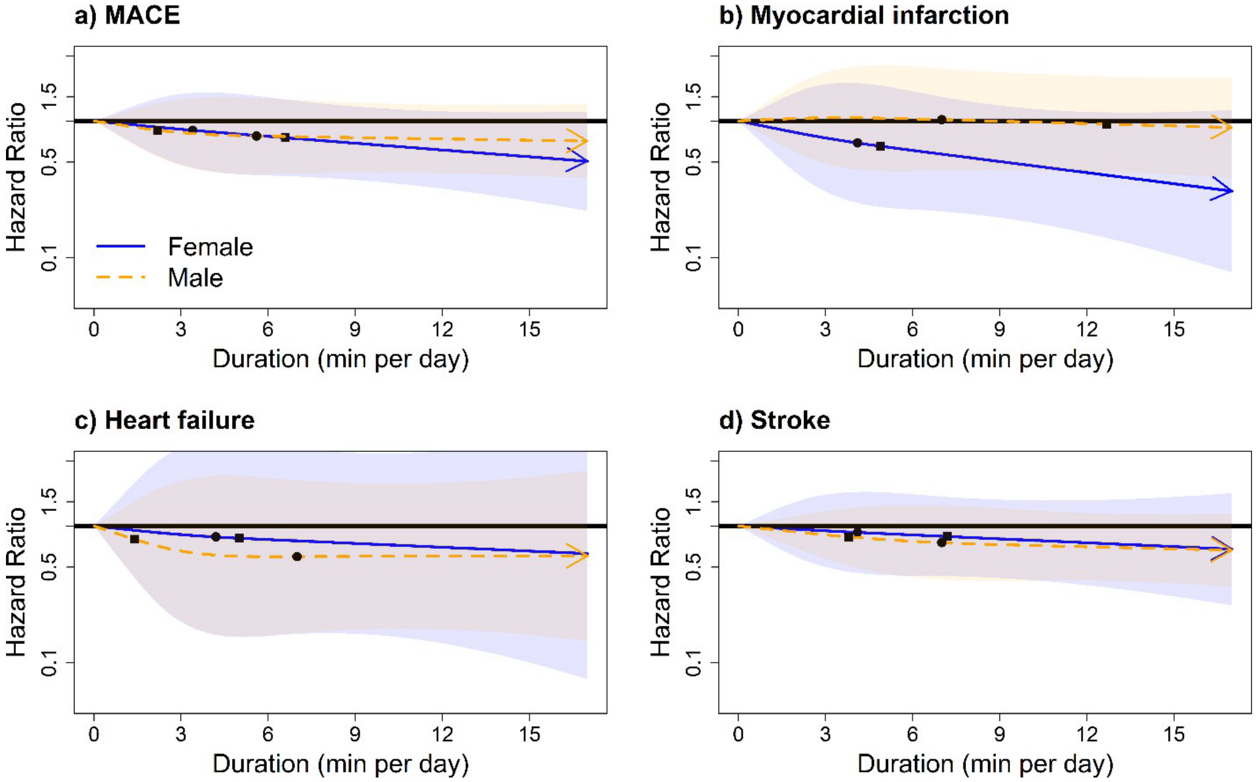

Legend: Adjusted for age, light intensity, moderate intensity, VILPA bouts over 1-minute, smoking history, alcohol consumption, accelerometry-derived sleep duration, diet, education, self-reported parental history of CVD, previous incidence of cancer, and self-reported medication use (cholesterol, blood pressure, and diabet es). **Panel A:** all MACE: n = 10,695; events: 201 (female/male = 79/122), **Panel B:** myocardial infarction: n = 10,601; events = 107 (female/male = 33/74). **Panel C:** heart failure: n = 10,531; events = 37 (female/male = 13/24). **Panel D:** stroke: n = 10,551; events = 57 (female/male = 33/24). Diamond, minimal dose, as indica ted by the ED_{50} statistic which estimates the daily duration of VILPA associated with 50% of optimal risk re duction. Circle, HR associated with the median VILPA value.

B) Age above median (62.5 -78.6 years**)**

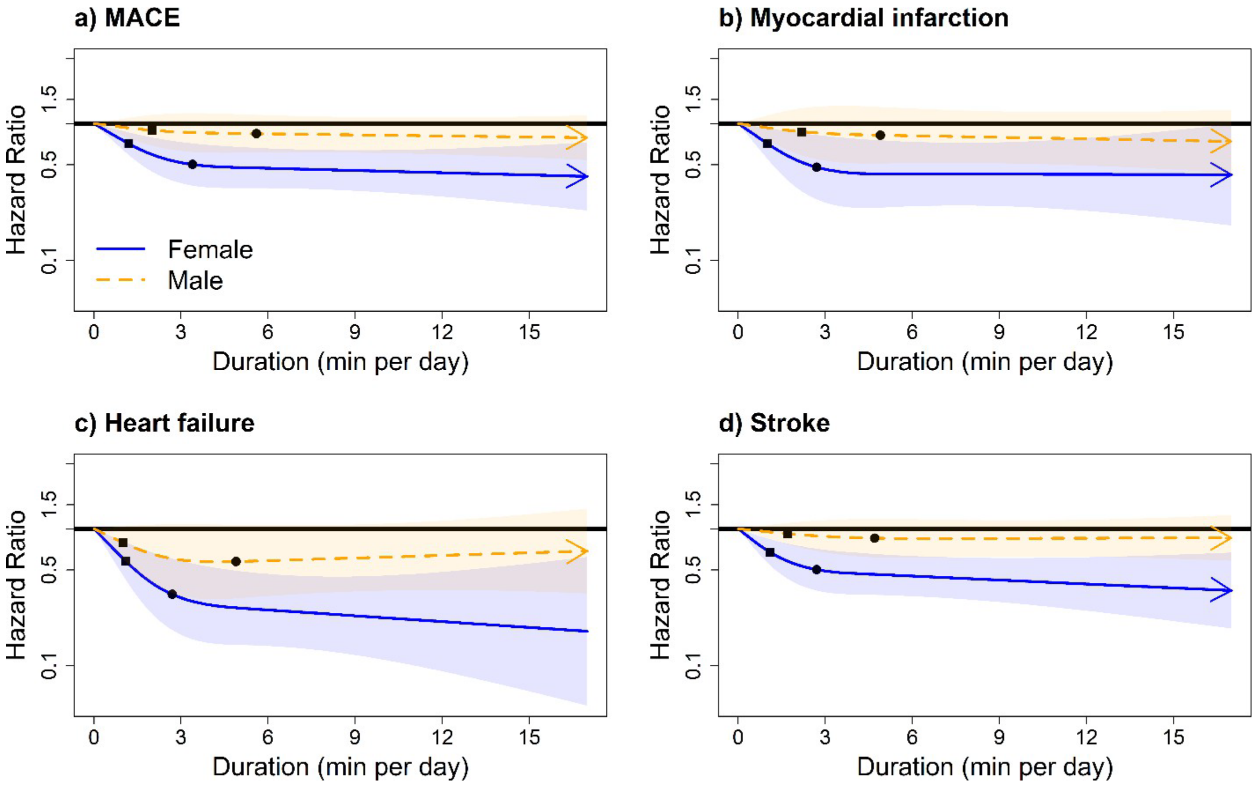

Legend: Adjusted for age, light intensity, moderate intensity, VILPA bouts over 1-minute, smoking history, alcohol consumption, accelerometry-derived sleep duration, diet, education, self-reported parental history of CVD, previous incidence of cancer, and self-reported medication use (cholesterol, blood pressure, and diabet es). **Panel A:** all MACE: n = 11,673; events: 618 (female/male = 252/366), **Panel B:** myocardial infarction: n = 11,327; events = 272 (female/male = 96/176). **Panel C:** heart failure: n = 10,602; events = 178 (female/m ale = 83/95). **Panel D:** stroke: n = 11,223; events = 168 (female/male = 73/95). Diamond, minimal dose, as i ndicated by the ED_{50} statistic which estimates the daily duration of VILPA associated with 50% of optimal ri sk reduction. Circle, HR associated with the median VILPA value.

eFigure 13: Adjusted Sex-specific dose response curves of light intensity physical activity for MACE and its subtypes for non-exercisers.

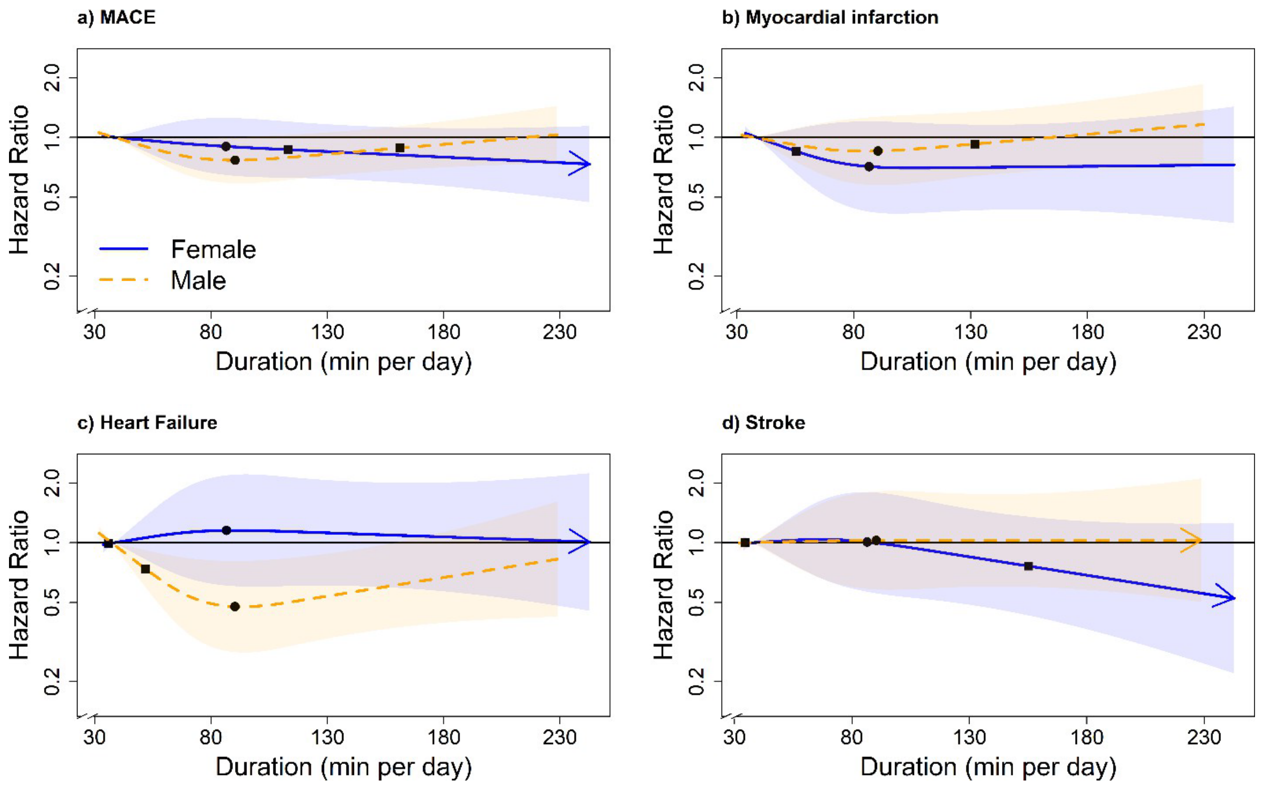

Legend: Adjusted for age, moderate intensity, vigorous intensity, smoking history, alcohol consumption, acc elerometry-derived sleep duration, diet, education, ethnicity, self-reported parental history of CVD, previous incidence of cancer, self-reported medication use (cholesterol, blood pressure, and diabetes) and residual of VILPA duration of 1-minute bouts. **Panel A:** all MACE: n = 22,368; events: 819 (female/male = 331/488), **P anel B:** myocardial infarction: n = 21,928; events = 379 (female/male = 129/250). **Panel C:** heart failure: n = 21,764; events = 215 (female/male = 96/119). **Panel D:** stroke: n = 21,774; events = 225 (female/male = 106 /119). Diamond, minimal dose, as indicated by the ED_{50} statistic which estimates the daily duration of VILP A associated with 50% of optimal risk reduction. Circle, HR associated with the median VILPA value.

eFigure 14: Adjusted Sex-specific dose response curves of moderate intensity physical activity for MACE and its subtypes for non-exercisers.

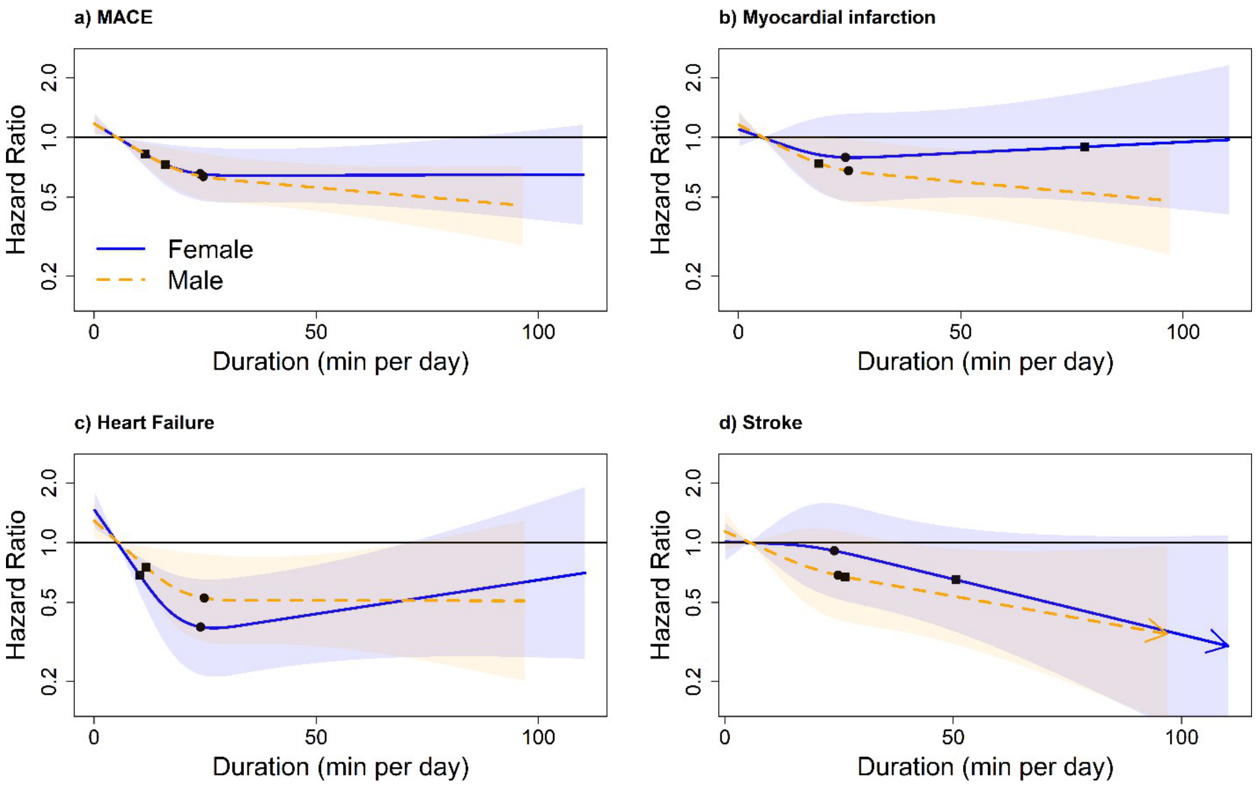

Legend: Adjusted for age, light intensity, vigorous intensity, smoking history, alcohol consumption, accelero metry-derived sleep duration, diet, education, ethnicity, self-reported parental history of CVD, previous incid ence of cancer, self-reported medication use (cholesterol, blood pressure, and diabetes) and residual of VILP A duration of 1-minute bouts. **Panel A:** all MACE: n = 22,368; events: 819 (female/male = 331/488), **Panel B:** myocardial infarction: n = 21,928; events = 379 (female/male = 129/250). **Panel C:** heart failure: n = 21,7 64; events = 215 (female/male = 96/119). **Panel D:** stroke: n = 21,774; events = 225 (female/male = 106/11 9). Diamond, minimal dose, as indicated by the ED_{50} statistic which estimates the daily duration of VILPA as sociated with 50% of optimal risk reduction. Circle, HR associated with the median VILPA value.

eFigure 15: Sex-specific adjusted dose response curves of daily VILPA duration for MACE and its sub-types, bouts lasting up to 1 minute (minutes/day) using cause-specific hazard models.

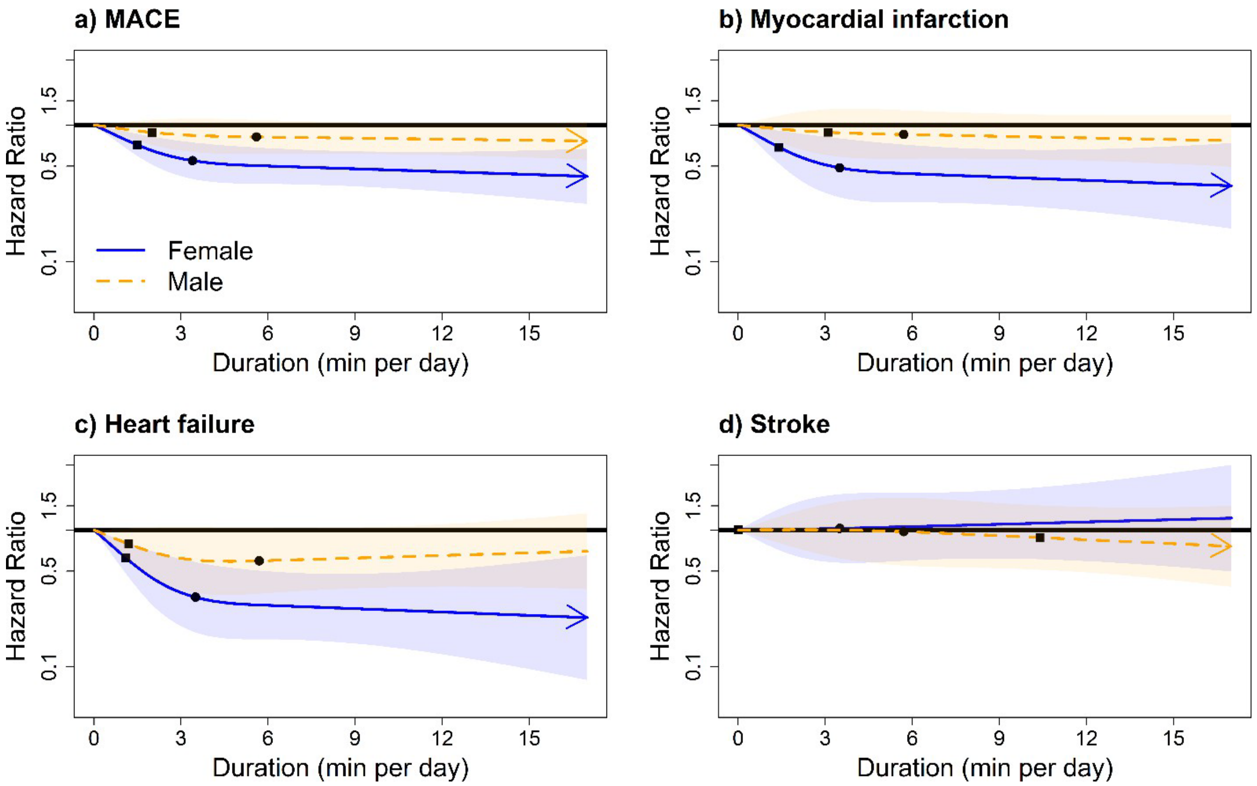

Legend: Adjusted for age, light intensity, moderate intensity, VILPA bouts over 1-minute, smoking history, alcohol consumption, accelerometer estimated sleep duration, diet, education, ethnicity, self-reported parental history of CVD, previous incidence of cancer, and self-reported medication use (cholesterol, blood pressure, and diabetes). **Panel A:** all MACE: n = 22,368; events: 819 (female/male = 331/488), **Panel B**: myocardial infarction: n = 21,928; events = 379 (female/male = 129/250). **Panel C**: heart failure: n = 21,764; events = 215 (female/male = 96/119). **Panel D**: stroke: n = 21,774; events = 225 (female/male = 106/119). Diamond, minimal dose, as indicated by the ED50 statistic which estimates the daily duration of VILPA associated with 50% of optimal risk reduction. Circle, HR associated with the median VILPA value.

eFigure 16: Adjusted Sex-specific dose response curves for MACE and its subtypes by length-standardized VILPA frequency; bouts lasting up to 1 min (bouts/day) using cause-specific hazard models.

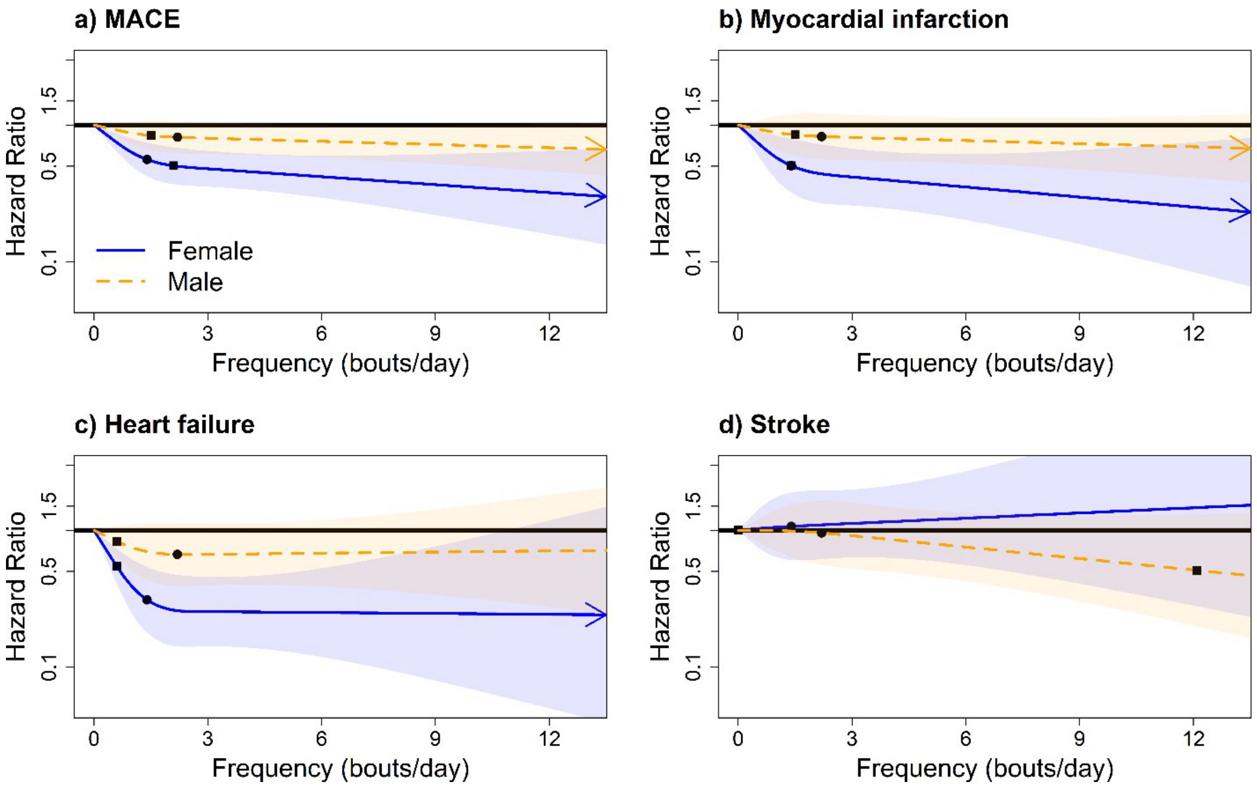

Legend: Adjusted for age, light intensity, moderate intensity, VILPA bouts over 1-minute, smoking history, alcohol consumption, accelerometer estimated sleep duration, diet, education, ethnicity, self-reported parental history of CVD, previous incidence of cancer, and self-reported medication use (cholesterol, blood pressure, and diabetes). The range was capped at the 97.5 percentile to minimize the influence of sparse data. **Panel A**: all MACE: n = 22,368; events: 819, **Panel B**: myocardial infarction: n = 21,928; events = 379. **Panel C**: heart failure: n = 21,764; events = 215. **Panel D**: stroke: n = 21,774; events = 225. Diamond, minimal dose, as indicated by the ED50 statistic which estimates the daily duration of VILPA associated with 50% of optimal risk reduction. Circle, HR associated with the median VILPA value.

eFigure 17: Adjusted sex-specific dose response curves of vigorous physical activity (VPA) in exercisers for MACE and its subtypes, bouts lasting up to 1 minute (minutes/day) using cause-specific hazard models.

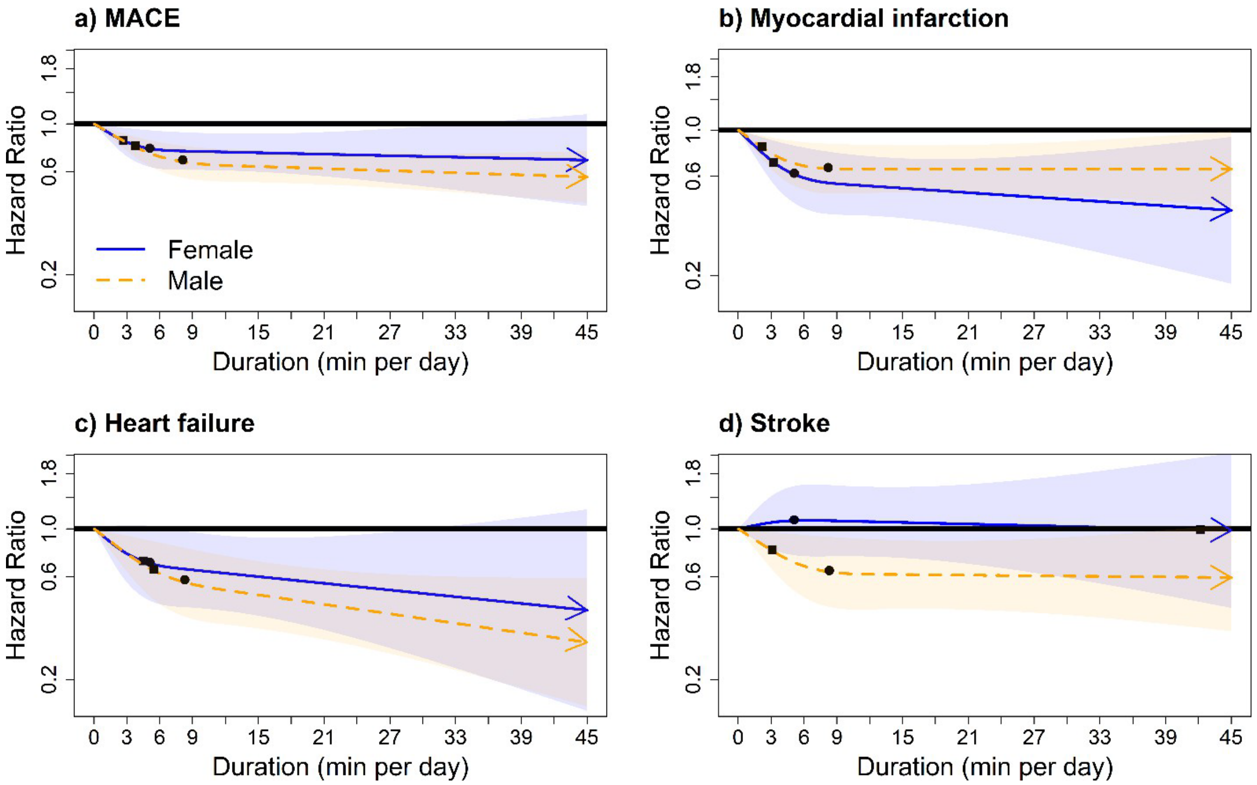

Legend: Adjusted for sex, age, light intensity, moderate intensity, VILPA bouts over 1-minute, smoking history, alcohol consumption, accelerometer estimated sleep duration, diet, education, ethnicity, self-reported parental history of CVD, previous incidence of cancer, and self-reported medication use (cholesterol, blood pressure, and diabetes). The range was capped at the 97.5 percentile to minimize the influence of sparse data. **Panel A**: MACE: n = 58,648; events: 1854 (female/male = 749/1105), **Panel B**: myocardial infarction: n = 57,622; events = 828 (female/male = 287/541). **Panel C**: heart failure: n = 57,289; events = 495 (female/male = 210/285). **Panel D**: stroke: n = 57,325; events = 531 (female/male = 252/279). Diamond, minimal dose, as indicated by the ED50 statistic which estimates the daily duration of VILPA associated with 50% of optimal risk reduction. Circle, HR associated with the median VILPA value.

eFigure 18: Sex-specific adjusted dose response curves of daily VILPA duration for MACE and its sub-types, bouts lasting up to 1 minute (minutes/day) where VILPA cut off is 7 METs.

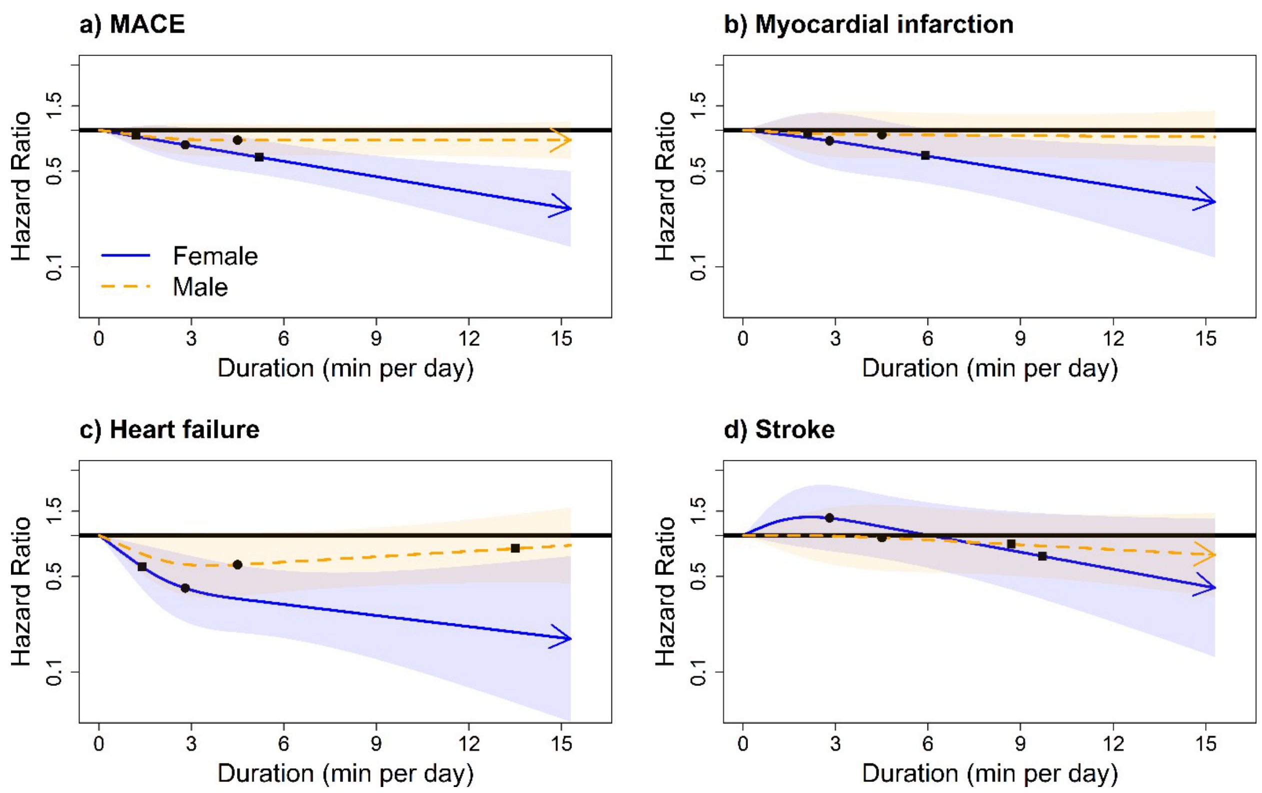

Legend: Adjusted for sex, age, light intensity, moderate intensity, VILPA bouts over 1-minute, smoking history, alcohol consumption, accelerometer estimated sleep duration, diet, education, ethnicity, self-reported parental history of CVD, previous incidence of cancer, and self-reported medication use (cholesterol, blood pressure, and diabetes). **Panel A**: MACE: n = 58,648; events: 1854 (female/male = 749/1105), **Panel B**: myocardial infarction: n = 57,622; events = 828 (female/male = 287/541). **Panel C**: heart failure: n = 57,289; events = 495 (female/male = 210/285). **Panel D**: stroke: n = 57,325; events = 531 (female/male = 252/279). Diamond, minimal dose, as indicated by the ED50 statistic which estimates the daily duration of VILPA associated with 50% of optimal risk reduction. Circle, HR associated with the median VILPA value.

eFigure 19: Sex-specific adjusted dose response curves of daily VILPA duration for MACE and its sub-types, bouts lasting up to 1 minute (minutes/day) where VILPA cut off is 8 METs.

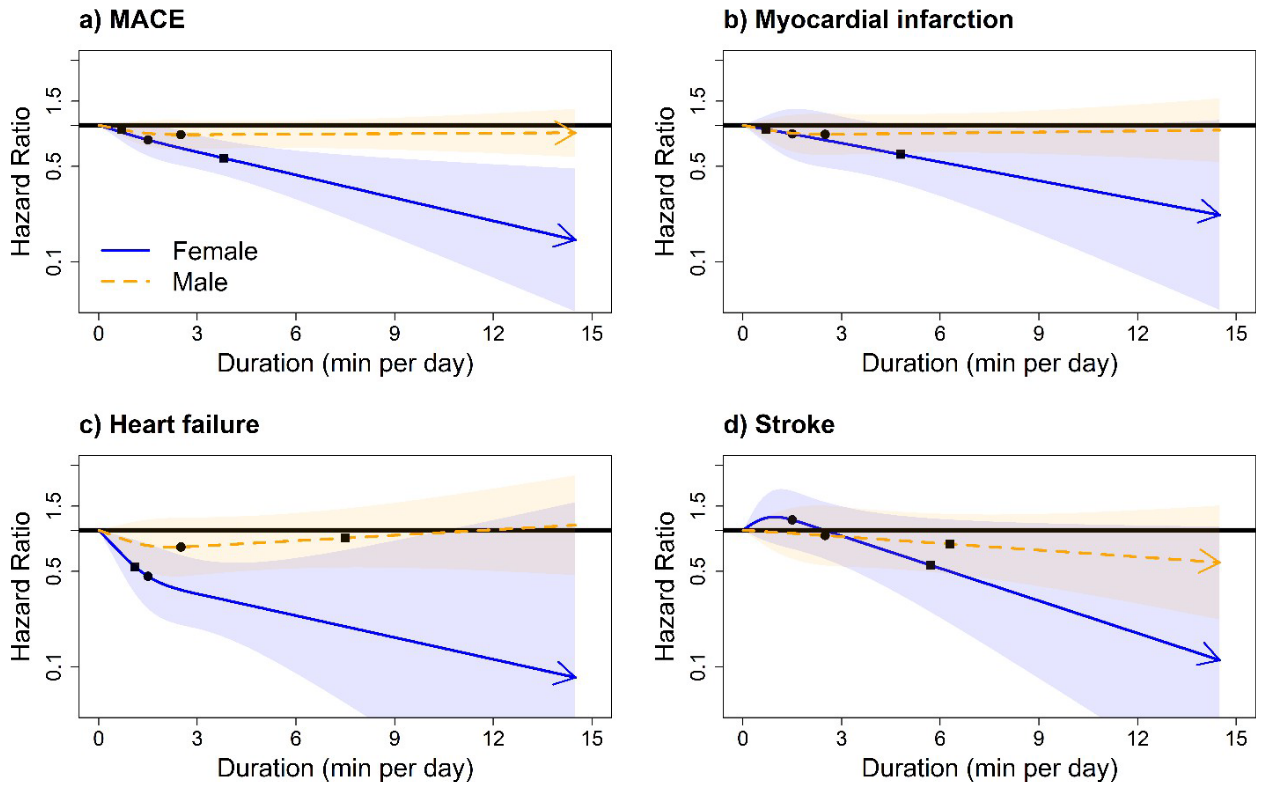

Legend: Adjusted for sex, age, light intensity, moderate intensity, VILPA bouts over 1-minute, smoking history, alcohol consumption, accelerometer estimated sleep duration, diet, education, ethnicity, self-reported parental history of CVD, previous incidence of cancer, and self-reported medication use (cholesterol, blood pressure, and diabetes). **Panel A**: MACE: n = 58,648; events: 1854 (female/male = 749/1105), **Panel B**: myocardial infarction: n = 57,622; events = 828 (female/male = 287/541). **Panel C**: heart failure: n = 57,289; events = 495 (female/male = 210/285). **Panel D**: stroke: n = 57,325; events = 531 (female/male = 252/279). Diamond, minimal dose, as indicated by the ED50 statistic which estimates the daily duration of VILPA associated with 50% of optimal risk reduction. Circle, HR associated with the median VILPA value.

eFigure 20: Sex-specific adjusted dose response curves of daily VPA duration for MACE and its sub-types, bouts lasting up to 1 minute (minutes/day) where VPA cut off is 7 METs.

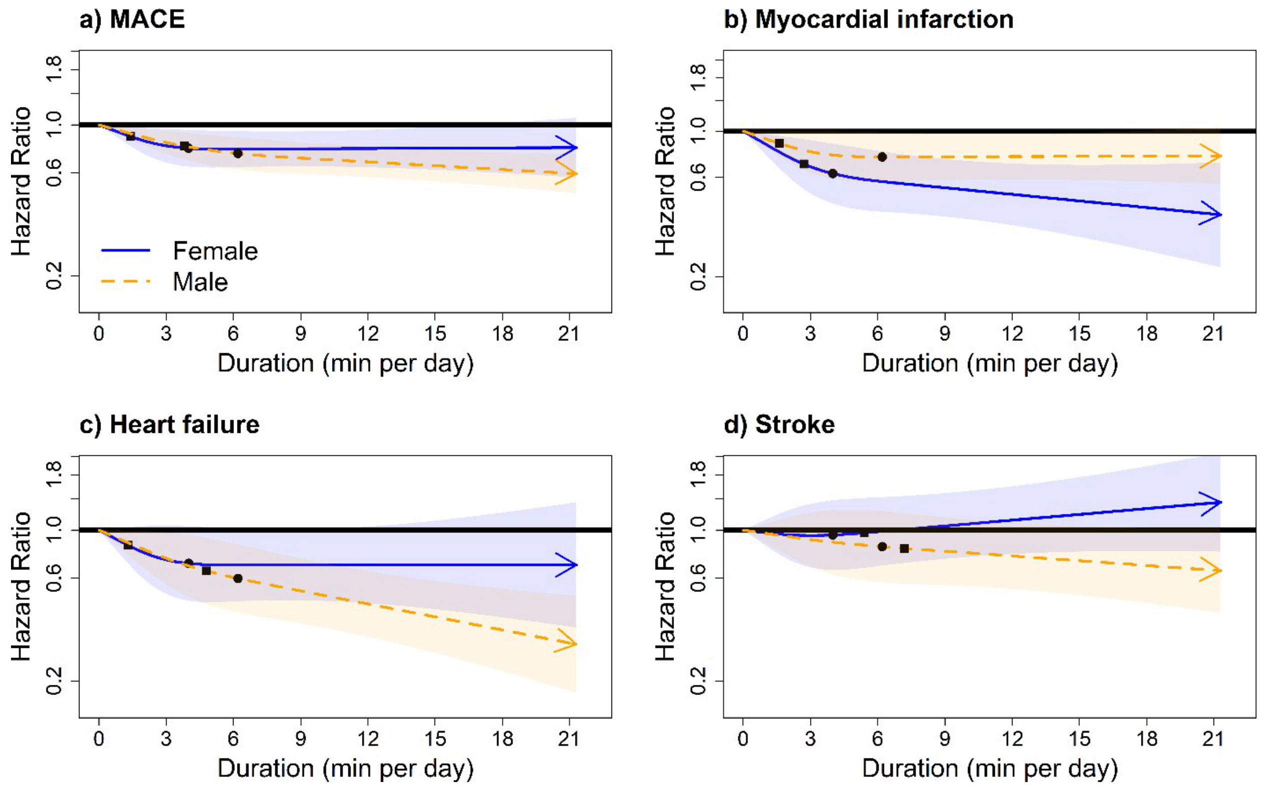

Legend: Adjusted for sex, age, light intensity, moderate intensity, VPA bouts over 1-minute, smoking history, alcohol consumption, accelerometer estimated sleep duration, diet, education, ethnicity, self-reported parental history of CVD, previous incidence of cancer, and self-reported medication use (cholesterol, blood pressure, and diabetes). **Panel A**: MACE: n = 58,648; events: 1854 (female/male = 749/1105), **Panel B**: myocardial infarction: n = 57,622; events = 828 (female/male = 287/541). **Panel C**: heart failure: n = 57,289; events = 495 (female/male = 210/285). **Panel D**: stroke: n = 57,325; events = 531 (female/male = 252/279). Diamond, minimal dose, as indicated by the ED50 statistic which estimates the daily duration of VILPA associated with 50% of optimal risk reduction. Circle, HR associated with the median VILPA value.

eFigure 21: Sex-specific adjusted dose response curves of daily VILPA duration for MACE and its sub-types, bouts lasting up to 1 minute (minutes/day) where VPA cut off is 8 METs.

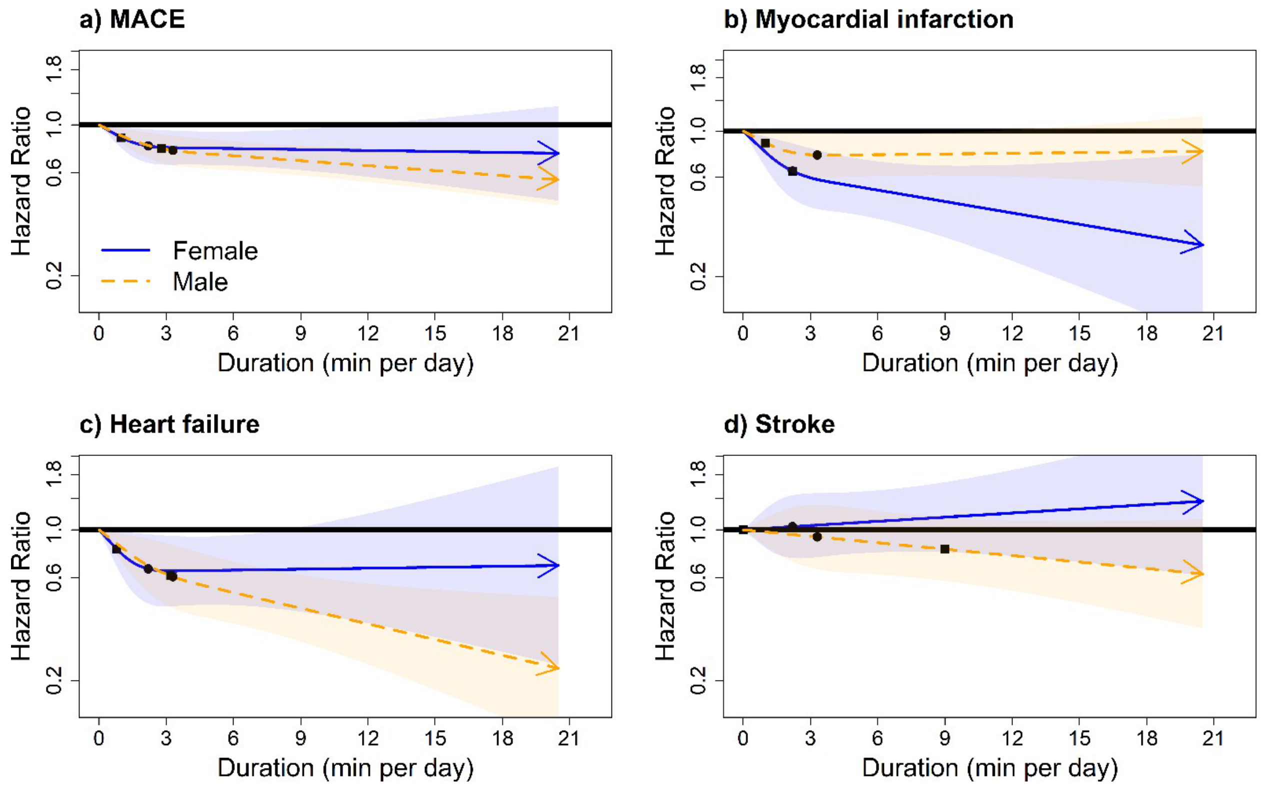

Legend: Adjusted for sex, age, light intensity, moderate intensity, VPA bouts over 1-minute, smoking history, alcohol consumption, accelerometer estimated sleep duration, diet, education, ethnicity, self-reported parental history of CVD, previous incidence of cancer, and self-reported medication use (cholesterol, blood pressure, and diabetes). **Panel A**: MACE: n = 58,648; events: 1854 (female/male = 749/1105), **Panel B**: myocardial infarction: n = 57,622; events = 828 (female/male = 287/541). **Panel C**: heart failure: n = 57,289; events = 495 (female/male = 210/285). **Panel D**: stroke: n = 57,325; events = 531 (female/male = 252/279). Diamond, minimal dose, as indicated by the ED50 statistic which estimates the daily duration of VILPA associated with 50% of optimal risk reduction. Circle, HR associated with the median VILPA value.

eText 1: Physical activity classification

Physical activity was classified using a previously validated wrist-worn accelerometery Random Forest (RF) activity classifier.[1] RF is an ensemble of multiple decision trees. Each tree is learned on a bootstrap sample of training data and each node in the tree is split using the best among a randomly selected set of acceleration features. The decisions from each tree are aggregated and a final model prediction is based on majority vote. The RF model requires very little pre-processing of the data, as the features do not need to be normalized. Additionally, the model is resistant to over fitting the training data because each tree within the forest is independently grown to maximum depth using a randomly selected subset of features.

The classifier categorized physical activity in 10 second windows into 1 of 4 activity classes: sedentary, standing utilitarian movements (ironing a shirt, washing dishes), walking (gardening, active commuting, mopping floors), and running/high energetic activities (active playing with children). These activities were then assigned to 1 of 4 activity intensities: sedentary, light, moderate, and vigorous. Walking activities were classified as light (an acceleration value of <100mg), moderate (≥100mg), and vigorous (≥400mg). The figure below depicts the activity classification scheme. Differentiation from sleep[2] and non-wear[3] was identified using the change in tilt angle and acceleration standard deviation. Monitors were calibrated[4] and corrected for orientation[5] using previously published methods.

Activity classification scheme

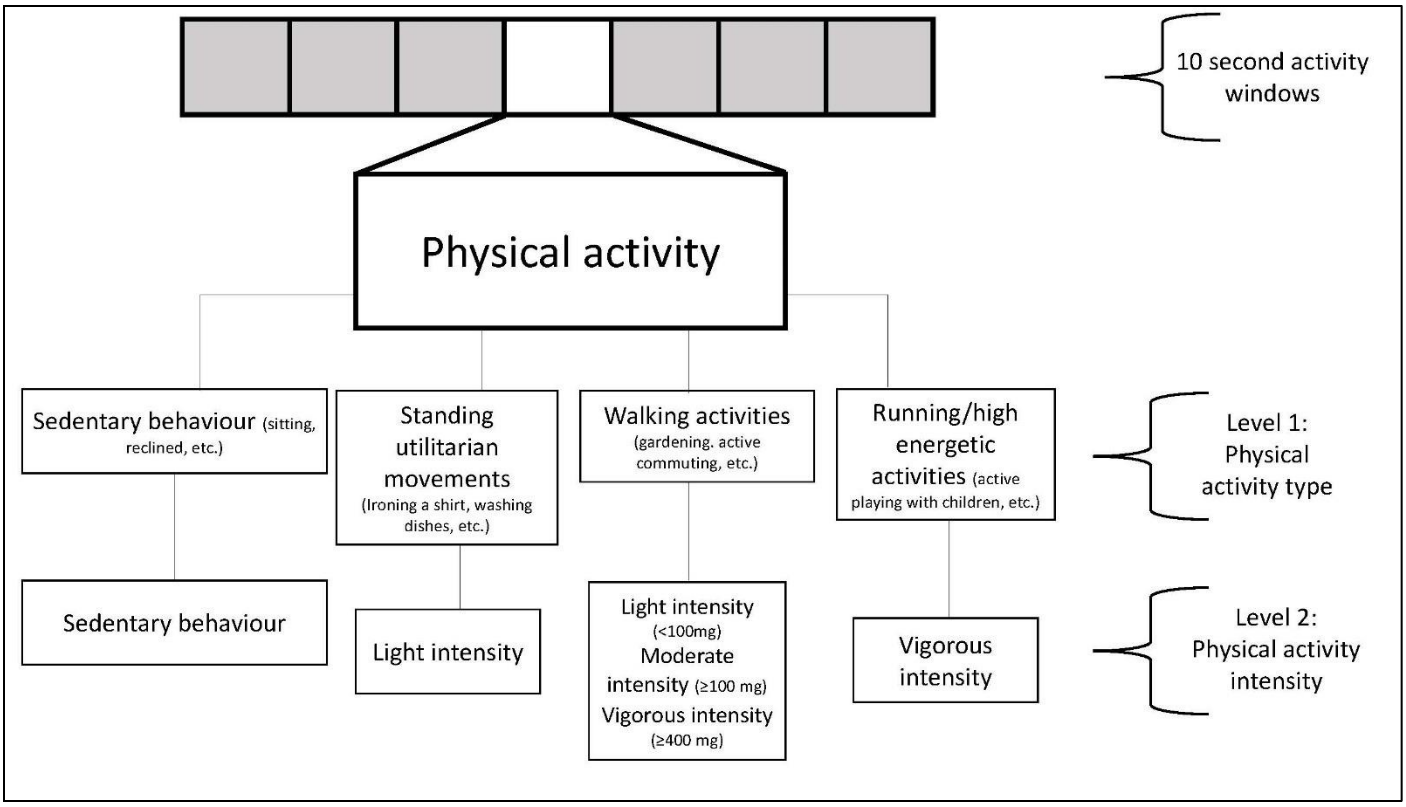

Activities in an independent sample of 102 participants (Age = 55.8 ± 12.4; 55.8% female) from the US[6] and Australia[7] (includes published and unpublished data) providing 105,767 activity samples from structured and free-living activities (17,627 minutes) were used to assess robustness and generalizability of the classifier. For free-living activities participant-worn or researcher-held Go-Pro video-recordings were used to attain ground-truth physical activity. Video files were imported into the Noldus Observer XT software for continuous direct observation coding. A two-stage direct observation scheme was implemented in which the participant's movement behaviour was coded for activity type and then activity intensity based on Compendium of Physical Activities[8]. The direct observation system generated a vector of date-time stamps corresponding to the start and finish of each movement event, which were used to assign the activity codes to the corresponding time segments of the accelerometer data. Interobserver reliability was assessed by dual coding. The intraclass correlation coefficient for coding activities was 0.912 (0.866-0.942). We present the classification and confusion matrix results below in **eText Tables 1 and 2**.

From a subset of the participants who had both ground-truth physical activity type-specific data ascertained through video recordings and the physical activity compendium, and physical activity intensity using indirect calorimetry (both gold-standard measures) we assessed intensity classification for individual activity types presented in the "expanded confusion matrix" below (**eText Table 3**; n=91; 245,945 seconds of activity data). Because of the >100 specific types of physical activity recorded we present the 3 most prevalent activity types for each intensity category.
We also provide a confusion matrix in **eText Table 4** below showing the performance among participants who self-reported as non-exercisers in the independent validity testing (n=88; 221,640 seconds of activity data).

Performance was further evaluated in a sample of 151 adults (age range 18-91 years, 65.6% female) recruited from the UK[9]. Participants in this dataset wore body cameras that provided pictures every 20 seconds to annotate ground-truth free-living activity labels. The picture-based activity coding scheme has been previously described[9]. Due to activities being coded once every 20 seconds, previously used methods in this dataset of consistent activity codes for at least 10 minutes were used to extract time segments. A total of 172,360 activity samples (28,727 minutes) were provided by participants. We present these results below in **eText Figure 1**.

Classifier performance in the three datasets is provided below:
eText Table 1: Intensity classification performance

	Sensitivity	Specificity	Precision	F-score	Overall Accuracy	Weighted Kappa	Overall F-score
Sedentary	86.5	93.7	90.5	88.5			
Light	71.2	89.4	55.8	62.6			
Moderate	85.4	96.6	92.7	88.9			
Vigorous	95.4	99.4	94.6	95.0			
					84.6	0.78	83.8

eText Table 2: Confusion matrix

	Sedentary	Light	Moderate	Vigorous
Sedentary	**36,904**	5,232	508	2
Light	3,120	**11,712**	1,612	17
Moderate	502	4,016	**29,528**	526
Vigorous	226	17	214	**9,470**

Rows= ground truth; columns=predictions; bold=correct labels; numbers represent each 10-second window; Derived from the US and Australian datasets

eText Table 3: Expanded confusion matrix of most prominent activity types within each intensity band in the independent validity testing (n=91; 245,945 seconds of activity data)

		Sedentary	Light	Moderate	Vigorous
**Sedentary**					
	Desk/computer work	**90.2%**	9.8%	-	-
	Sitting/lying	**93.9%**	6.1%	-	-
	Sitting using phone/appliance	**89.4%**	10.6%	-	-
**Light**					
	Washing dishes/kitchen activities	17.3%	**80.8%**	2.9%	-
	Household chores standing	12.5%	**83.6%**	3.9%	-
	slow walking/grocery shopping	15.2%	**77.8%**	7.0%	-
**Moderate**					
	Walking briskly/fast (e.g. for transportation)	-	11.4%	**86.2%**	2.4%
	Household chores cleaning rooms/ambulation	-	12.2%	**87.6%**	0.2%
	Occupation brisk walking carrying light objects	-	11.8%	**87.5%**	0.7%
**Vigorous**					
	Manual work	-	3.0%	1.3%	**95.7%**
	Very fast walking/burst of running (e.g, for transportation)	-	0.8%	2.3%	**96.9%**
	Heavy household outdoor chores	-	2.4	4.2%	**93.4%**

Rows= ground truth; columns=predictions; bold=correct classification

eText Table 4a: Confusion matrix among female participants who identified as non-exercisers in the independent validity testing (n=44; 115,142 seconds of activity data)

	Sedentary	Light	Moderate	Vigorous
Sedentary	**92.6**	6.9	0.5	-
Light	15.4	**78.5**	5.8	0.3
Moderate	0.6	13.4	**85.4**	0.6
Vigorous	-	1.2%	1.9%	**96.9%**

Rows= ground truth; columns=predictions; bold=correct classification;

eText Table 4b: Confusion matrix among male participants who identified as non-exercisers in the independent validity testing (n=44; 106,498 seconds of activity data)

	Sedentary	Light	Moderate	Vigorous
Sedentary	**89.9**	9.7	0.4	-
Light	14.2	**79.2**	6.1	0.5
Moderate	0.6	11.1	**87.4**	0.9
Vigorous	-	2.5	2.9	**94.6**

Rows= ground truth; columns=predictions; bold=correct classification;

eText Figure 1: Participant-level specific sensitivity and precision in the UK sample. Error bars refer to 95% confidence intervals.

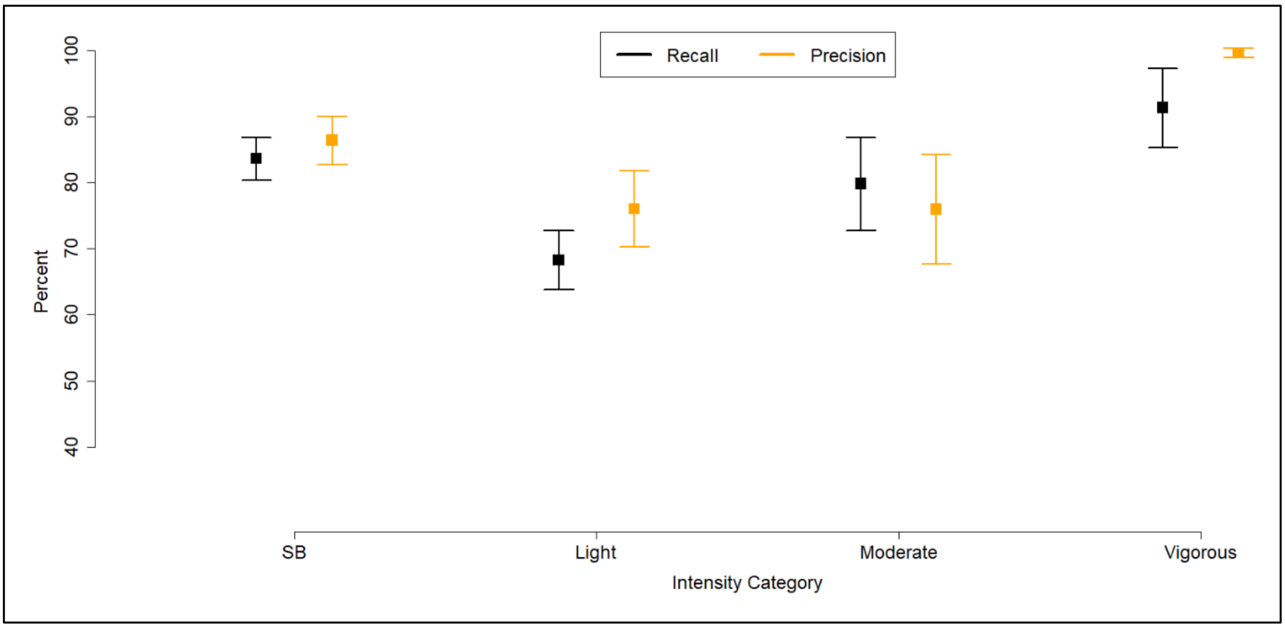

eText 1 References:

1. Pavey TG, Gilson ND, Gomersall SR, Clark B, Trost SG. Field evaluation of a random forest activity classifier for wrist-worn accelerometer data. *J Sci Med Sport*. 2017;20(1):75-80. doi:10.1016/j.jsams.2016.06.003
2. Van Hees VT, Sabia S, Jones SE, et al. Estimating sleep parameters using an accelerometer without sleep diary. *Sci Rep*. 2018;8(1):1-11. doi:10.1038/s41598-018-31266-z
3. Ahmadi MN, Nathan N, Sutherland R, Wolfenden L, Trost SG. Non-wear or sleep? Evaluation of five non-wear detection algorithms for raw accelerometer data. *J Sports Sci*. 2020;38(4). doi:10.1080/02640414.2019.1703301
4. Sipos M, Paces P, Rohac J, Novacek P. Analyses of Triaxial Accelerometer Calibration Algorithms. *IEEE Sens J*. 2011;12(5):1157-1165. doi:10.1109/jsen.2011.2167319
5. Mizell D. Using gravity to estimate accelerometer orientation. Paper presented at the Proceedings of the 7th IEEE International Symposium on Wearable Computers, White Plains, NY. doi:10.1515/zna-1966-0711
6. Reiss A, Weber M, Stricker D. Exploring and extending the boundaries of physical activity recognition. In: *IEEE International Conference on Systems, Man, and Cybernetics*. ; 2011:46-50. doi:10.1109/ICSMC.2011.6083640
7. Clark BK, Winkler EA, Brakenridge CL, Trost SG, Healy GN. Using bluetooth proximity sensing to determine where office workers spend time at work. *PLoS One*. 2018;13(3):1-15. doi:10.1371/journal.pone.0193971
8. Ainsworth BE, Haskell WL, Herrmann SD, et al. 2011 compendium of physical activities: A second update of codes and MET values. *Med Sci Sports Exerc*. 2011;43(8):1575-1581. doi:10.1249/MSS.0b013e31821ece12
9. Willetts M, Hollowell S, Aslett L, Holmes C, Doherty A. Statistical machine learning of sleep and physical activity phenotypes from sensor data in 96,220 UK Biobank participants. *Sci Rep*. 2018;8(7961). doi:10.1038/s41598-018-26174-1

eText 2: Bout length standardisation

As the length of raw bouts within the VILPA frequency exposure was highly variable, we length-standardized analytic bouts to one minute (for raw bouts lasting up to 1 minute) using a rolling sum on the time-series data until 1 or 2 minutes, respectively, was reached or exceeded. For example, a participant with five consecutive raw bouts lasting up to 1 minute each (20, 30, 20, 40, and 10 seconds long), would be assigned 1.83 analytic bouts: the first three raw bouts would count as one and the rolling sum would be reset; then the last two raw counts would count as 0.83 length-standardised bouts (50 seconds divided by 60). This bout handling has interpretational advantages as it permits a more concrete behavioural interpretation of the VILPA frequency findings than raw bouts, as each length-standardised bout can be specifically interpreted as lasting 1 minute.

eText 3: Physical activity energy expenditure, cardiorespiratory fitness, and relative physical activity intensity

In a sample of 2,043 female and 1,588 male non-exercisers with valid accelerometry data, we calculated physical activity energy expenditure during VILPA bouts using average acceleration (VO_2 = 0.0320 x average acceleration + 7.28)[1]. Cardiorespiratory fitness was measured using a 6-minute incremental ramp cycle ergometer test with workload calculated to age, height, weight, resting heart rate, and sex for each participant. Heart rate was measured using a four-lead ECG. Following established procedures[2] work rate at maximal heart rate was estimated by extrapolating pre-testing heart rate (work rate= 0 watts) and peak heart rate (peak work rate in watts) to age predicted maximal heart rate (max heart rate = 208 – 0.7 x age)[3]. Maximal oxygen consumption (VO_2max; ml/kg/min) was estimated for the relationship between work rate and oxygen uptake using the equation[4]: VO_2max = 7 + (10.8 x max work rate/ body mass (kg)).
Relative physical activity intensity (e.g., %VO2max) during VILPA bouts was calculated as the percentage of VO_2 expended relative to each participant. %VO2max higher than 64% is equivalent to vigorous intensity[5]. The results of these analyses are summarised in **eTable 11**.

eText 3 References

1. Hildebrand M, VT VANH, Hansen BH, Ekelund U. Age group comparability of raw accelerometer output from wrist- and hip-worn monitors. *Med Sci Sports Exerc.* 2014;46(9):1816-1824.
2. Celis-Morales CA, Lyall DM, Anderson J, et al. The association between physical activity and risk of mortality is modulated by grip strength and cardiorespiratory fitness: evidence from 498 135 UK-Biobank participants. *European heart journal.* 2017;38(2):116-122.
3. Tanaka H, Monahan KD, Seals DR. Age-predicted maximal heart rate revisited. *J Am Coll Cardiol.* 2001;37(1):153-156.
4. Swain DP. Energy cost calculations for exercise prescription: an update. *Sports Med.* 2000;30(1):17-22.
5. Garber CE, Blissmer B, Deschenes MR, et al. American College of Sports Medicine position stand. Quantity and quality of exercise for developing and maintaining cardiorespiratory, musculoskeletal, and neuromotor fitness in apparently healthy adults: guidance for prescribing exercise. *Med Sci Sports Exerc.* 2011;43(7):1334-1359.

eText 4: Unabridged sections of summarised results in main manuscript

Differences in the referent groups of females and males: Since very large differences in the characteristics of the referent group can lead to confounding by indication and spurious sex differences in dose-response, we compared health-related characteristics between females and males in the referent category (no VILPA) but we noted no systematic pattern of differences in favour of one sex group. For example, a higher proportion of males were smokers (19.4% vs. 11.6%) or were drinking above guidelines (43.4% vs. 21.6%) than females, while a lower proportion of females completed college education (34.7% vs 42.9%) or had a high fruit and vegetable consumption (26.3% vs 31.3%)

Exercisers sample: **eFigure 2** and **eTable 1** describe the sample derivation process and characteristics of the exercisers sample: over a mean follow-up of 7.9 (0.96) years (465,782 person-years) 58,648 exercisers were included in the all-MACE analyses (1,854 events; 749 female /1,105 male), *n*= 57,622 were included in the myocardial infarction analyses (828 events: 287 female / 541 male), *n* = 57,289 in the heart failure analyses (495 events: 210 female /285 male), and *n*=57,325 in the stroke analyses (531 events: 252 female /279 male).

eText 5: Directed acyclic graph (DAG) describing the theoretical causal and confounder pathways between VILPA and MACE.

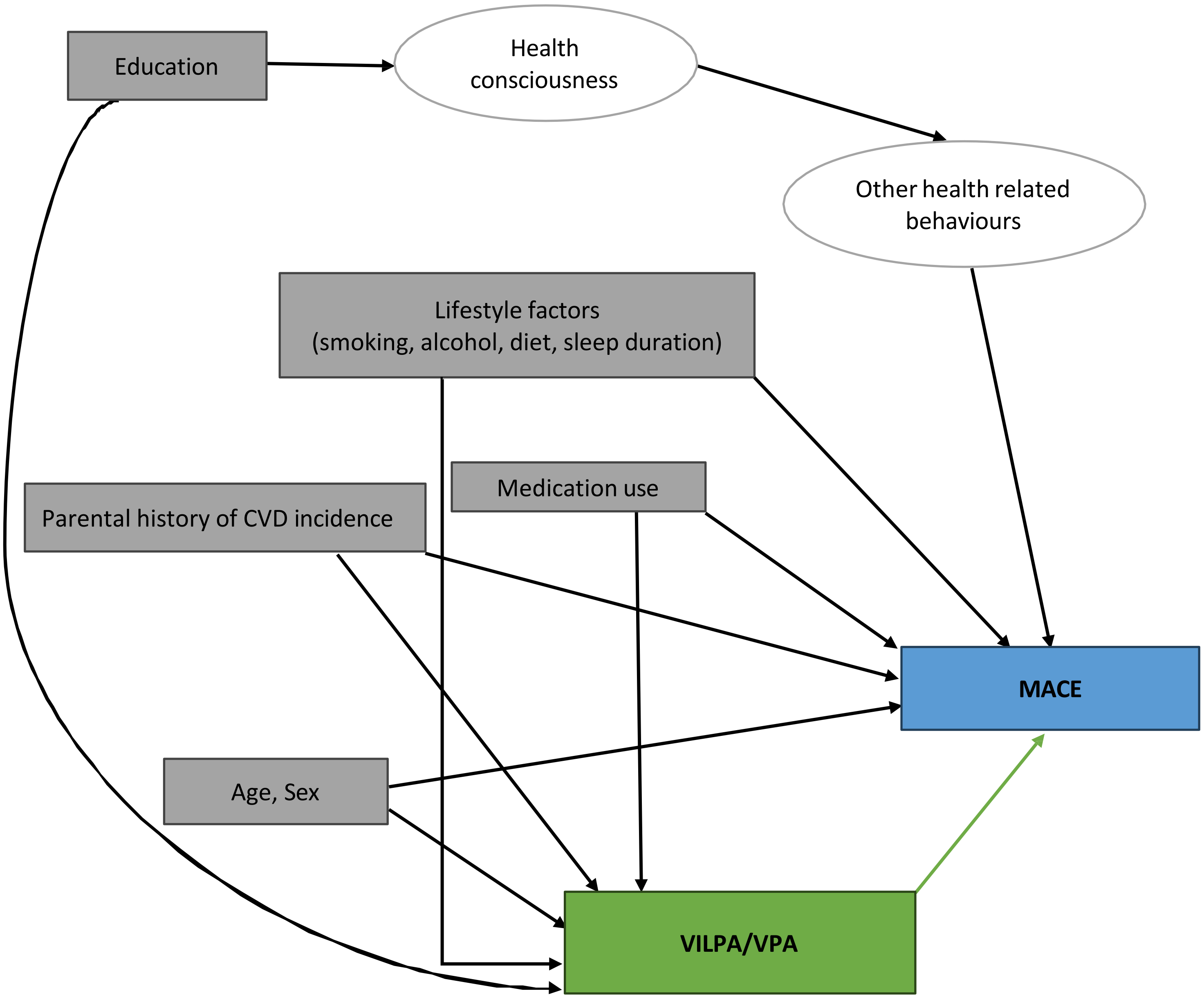

Green = exposure; blue = outcome; grey = confounders; white = unobserved variables

Bout length of VILPA (non-exercisers) and VPA (exercisers)

In the core MACE analyses sample of 22,368 participants, 89.1% of VILPA bouts lasted up to 1 minute and 92.9% lasted up to 2 minutes. The median VILPA daily duration was 4.3 minutes (3.4/5.6 minutes for females/males) per day. The median length-standardised frequency was 1.7 (females: 1.4; males: 2.2) bouts per day. The median daily raw frequency was 10.1 (females: 9.3; males: 11.4 bouts per day. Among the 58,648 exercisers entered in the comparative all-MACE analyses, the large majority of (context agnostic) VPA was accrued in bouts lasting up to one (85.3% of all VPA bouts) or two (92.6%) minutes. Median VPA daily duration was 6.1 (females: 5.1; males: 8.1).

Sensitivity analyses

All sensitivity analyses produced results consistent with the main findings (**eFigures 8-17**): adjusting for clinical factors that could be considered potential mediators of the association between VILPA and MACE (**eFigure 8**), excluding participants who self-reported poor health or were underweight or were current smokers (**eFigure 9**), using the 15th percentile of the VILPA distribution (0.63 minutes per day) as the referent data point (**eFigure 10**) or excluding frail participants (**eFigure 11**) produced very similar findings to the results of the main analyses presented in **Figure 2**.
